# *Ov*-RPA–CRISPR/Cas12a assay for the detection of *Opisthorchis viverrini* infection in field-collected human feces

**DOI:** 10.1186/s13071-024-06134-7

**Published:** 2024-02-21

**Authors:** Orawan Phuphisut, Akkarin Poodeepiyasawat, Tippayarat Yoonuan, Dorn Watthanakulpanich, Charin Thawornkuno, Onrapak Reamtong, Megumi Sato, Poom Adisakwattana

**Affiliations:** 1 Department of Helminthology, Faculty of Tropical Medicine, Mahidol University, Bangkok, 10400 Thailand; 2 Department of Molecular Tropical Medicine and Genetics, Faculty of Tropical Medicine, Mahidol University, Bangkok, 10400 Thailand; 3 Graduate School of Health Sciences, Niigata University, Niigata, Japan

**Keywords:** Clustered regularly interspaced short palindromic repeats (CRISPR)/CRISPR-associated protein 12a (CRISPR/Cas12a), Diagnostic method, Fecal sample, Opisthorchiasis, *Opisthorchis viverrini*, Recombinase polymerase amplification

## Abstract

**Background:**

*Opisthorchis viverrini* infection is traditionally diagnosed using the Kato–Katz method and formalin ethyl–acetate concentration technique. However, the limited sensitivity and specificity of these techniques have prompted the exploration of various molecular approaches, such as conventional polymerase chain reaction (PCR) and real-time PCR, to detect *O. viverrini* infection. Recently, a novel technique known as recombinase polymerase amplification (RPA)–clustered regularly interspaced short palindromic repeats (CRISPR)/CRISPR-associated protein (Cas) (RPA–CRISPR/Cas) assay was developed as a point-of-care tool for the detection of various pathogens, including viruses and bacteria such as severe acute respiratory syndrome coronavirus 2 and *Mycobacterium tuberculosis*. This technology has demonstrated high sensitivity and specificity. Therefore, we developed and used the RPA–CRISPR/Cas assay to detect *O. viverrini* infection in field-collected human feces.

**Methods:**

To detect *O. viverrini* infection in fecal samples, we developed a CRISPR/Cas12a (RNA-guided endonuclease) system combined with RPA (*Ov*-RPA–CRISPR/Cas12a). Several fecal samples, both helminth-positive and helminth-negative, were used for the development and optimization of amplification conditions, CRISPR/Cas detection conditions, detection limits, and specificity of the RPA–CRISPR/Cas12a assay for detecting *O. viverrini* infection. The detection results were determined using a real-time PCR system based on fluorescence values. Additionally, as the reporter was labeled with fluorescein, the detection results were visually inspected using an ultraviolet (UV) transilluminator. A receiver operating characteristic curve (ROC) was used to determine the optimal cutoff value for fluorescence detection. The diagnostic performance, including sensitivity and specificity, of the *Ov*-RPA–CRISPR/Cas12a assay was evaluated on the basis of comparison with standard methods.

**Results:**

The *Ov*-RPA–CRISPR/Cas12a assay exhibited high specificity for detecting *O. viverrini* DNA. On the basis of the detection limit, the assay could detect *O. viverrini* DNA at concentrations as low as 10^−1^ ng using the real-time PCR system. However, in this method, visual inspection under UV light required a minimum concentration of 1 ng. To validate the *Ov*-RPA–CRISPR/Cas12a assay, 121 field-collected fecal samples were analyzed. Microscopic examination revealed that 29 samples were positive for *O. viverrini*-like eggs. Of these, 18 were confirmed as true positives on the basis of the *Ov*-RPA–CRISPR/Cas12a assay and microscopic examination, whereas 11 samples were determined as positive solely via microscopic examination, indicating the possibility of other minute intestinal fluke infections.

**Conclusions:**

The *Ov*-RPA–CRISPR/Cas12a assay developed in this study can successfully detect *O. viverrini* infection in field-collected feces. Due to the high specificity of the assay reported in this study, it can be used as an alternative approach to confirm *O. viverrini* infection, marking an initial step in the development of point-of-care diagnosis.

**Graphical abstract:**

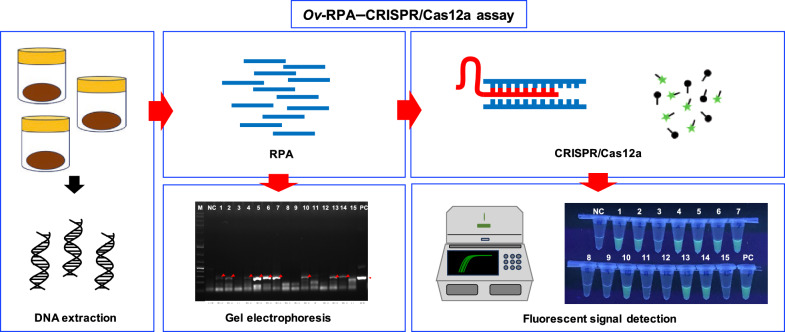

**Supplementary Information:**

The online version contains supplementary material available at 10.1186/s13071-024-06134-7.

## Background

The liver fluke *Opisthorchis viverrini* is the causative agent of opisthorchiasis [1, 2]. Opisthorchiasis presents a major public health concern in Southeast Asia, particularly in Thailand, the Lao People’s Democratic Republic, Cambodia, and central Vietnam [3, 4]. Conventional standard methods for the diagnosis of *O. viverrini* infection include the Kato–Katz (KK) method or formalin ethyl–acetate concentration technique (FECT), both of which detect eggs in feces [5, 6]. Despite being reported as the most sensitive method [7], FECT has proven unreliable for detecting mild infection (< 20 worms) [8, 9]. Moreover, the egg morphology of *O. viverrini* is comparable to that of minute intestinal flukes, including *Haplorchis taichui* and *H. pumilio*, which often coexist in the same endemic area, complicating species differentiation [10–12].

To address these challenges in detecting *O. viverrini* infection in fecal samples, molecular assays have been developed using various platforms, including conventional polymerase chain reaction (PCR) [13–18], TaqMan real-time PCR [19, 20], and loop-mediated isothermal amplification [21, 22]. Although these assays demonstrate high sensitivity and specificity, some techniques, particularly PCR-based methods, require specialized equipment that is impractical for field use. Recently, the application of the CRISPR/Cas system, an adaptive immune system found in archaea and bacteria [23], has facilitated the detection of various pathogens, including viruses and bacteria. The CRISPR/Cas system has previously been used to detect various pathogens, such as Zika virus [24], avian influenza A (H7N9) virus [25], Ebola virus [26], plasma Epstein–Barr virus DNA [27], severe acute respiratory syndrome coronavirus 2 [28], *Staphylococcus aureus* [29], *Mycobacterium tuberculosis* [30], and *Enterocytozoon hepatopenaei* [31]. Importantly, when combined with isothermal amplification techniques, such as recombinase polymerase amplification (RPA), the CRISPR/Cas system can detect target nucleic acid molecules with attomolar (aM) level sensitivity. Moreover, as RPA and CRISPR/Cas system do not require temperature cycling, the RPA–CRISPR/Cas assay can be performed using only basic equipment [32, 33]. Labeling reporters with fluorescein produces fluorescent signals that can be visually inspected using light-emitting diode (LED) or ultraviolet (UV) light. Additionally, the detection result of the CRISPR/Cas system can be read using the lateral flow strip method. This indicates the potential development of this assay into a field-deployable method [34, 35]. However, the CRISPR/Cas system has not previously been used to detect *O. viverrini* infection.

In this study, a combination of CRISPR/Cas12a (an RNA-guided endonuclease) system and RPA was used to detect *O. viverrini* infection in fecal samples. The detection results were determined using a real-time PCR system based on fluorescence values. Additionally, as the reporter was labeled with fluorescein, the detection results were visually inspected using an UV transilluminator. The target gene for detecting *O. viverrini* infection was the NADH dehydrogenase subunit 1 (*NAD1*) gene. The diagnostic performance, including sensitivity and specificity, of the *Ov*-RPA–CRISPR/Cas12a assay was evaluated on the basis of comparison with standard methods (KK and FECT).

## Methods

### Optimization of the *Ov*-RPA–CRISPR/Cas12a assay in the laboratory

#### Oligonucleotide primers and single-guide RNA (sgRNA) design

On the basis of molecular detection results from previous studies, *NAD1*, which is encoded in high copy numbers by the mitochondrial genome, has been identified as a potential target region for detecting *O. viverrini* infection [18, 21, 36, 37]. Furthermore, established DNA sequences from various parasitic helminths are available, facilitating the development of primers and probes specifically directed against *O. viverrini* DNA [38].

To establish the *Ov*-RPA–CRISPR/Cas12a assay, oligonucleotide primers and sgRNA were designed and developed. The nucleotide sequences used to develop the oligonucleotide primers are provided in Additional file 1: Table S1A. The primer pair for *O. viverrini* was manually selected at a specific site. The oligonucleotide primers and sgRNA were designed following the manufacturer’s instructions of TwistDx RPA (TwistDx Limited, Maidenhead, UK) and EnGen® Lba Cas12a (Cpf1) (New England Biolabs, Ipswich, MA, USA), respectively. Multiple sequence alignment revealed the exact locations of the oligonucleotide primers and sgRNA (Additional file 2: Fig. S1). The oligonucleotide primers and sgRNA used for the *Ov*-RPA–CRISPR/Cas12a assay are shown in Additional file 1: Table S1B. A single-stranded DNA (ssDNA) reporter sequence was used on the basis of the study by Chen et al. [39] (Additional file 1: Table S1B). Before further investigation, the specificity of the oligonucleotide primers and sgRNA was confirmed by blasting them against a nucleotide database of *Homo sapiens* (taxid: 9606), nematodes (taxid: 6231), and platyhelminths (taxid: 6157) using Primer-BLAST (https://www.ncbi.nlm.nih.gov/tools/primer-blast/).

To examine the quality of DNA in the extracted fecal samples (copro-DNA), the human actin gene (*hACTB*) was used as an internal control. The oligonucleotide primers were generated using Primer3Plus (https://www.primer3plus.com) following the manufacturer’s instructions (TwistDx RPA). To test for the presence of any inhibitory factors in the extracted fecal samples, RPA was conducted by equally spiking pET20b^+^ vector containing a DNA template for the *Gnathostoma spinigerum* carboxypeptidase C2 gene (pET20b^+^–*GsCPC2*) into copro-DNA samples. The developed *Ov*-RPA–CRISPR/Cas12a assay was limited to samples in which *hACTB* and *GsCPC2* amplicons were detected. All oligonucleotide primers used in this study are listed in Additional file 1: Table S1B.

#### *O. viverrini* and other helminths’ genomic DNA controls

To evaluate the specificity of oligonucleotide primers for RPA, we used the genomic DNA of helminths other than *O. viverrini*—including *Ascaris lumbricoides*, *Trichuris trichiura*, *Necator americanus*, *Haplorchis taichui*, *Clonorchis sinensis*, and *Fasciola gigantica*, which are found in the gastrointestinal tract and liver bile duct—as DNA templates for the *Ov*-RPA–CRISPR/Cas12a assay. We extracted the DNA of *A. lumbricoides*, *T. trichiura*, *N. americanus*, *H. taichui*, *C. sinensis*, *F. gigantica*, and *O. viverrini* adult worms using Tissue Genomic DNA Mini Kit (Geneaid Biotech Ltd., New Taipei City, Taiwan) following the manufacturer’s instructions. We determined the quality and quantity of DNA samples using NanoDrop (Thermo Fisher Scientific Inc., Waltham, MA, USA) and 1% agarose gel electrophoresis.

A plasmid containing the *O. viverrini* *NAD1* gene (pGEMT–*OvNAD1*) was used as a positive control for the *Ov*-RPA–CRISPR/Cas12a assay. We constructed the pGEMT–*OvNAD1* plasmid following the manufacturer’s instructions (Promega Corporation, Madison, WI, USA). Briefly, we amplified the *NAD1* amplicon using *O. viverrini* DNA as the template via Taq DNA polymerase (Thermo Fisher Scientific, Inc.). Subsequently, the amplicon was ligated into the pGEM-T Easy vector and then transformed into *Escherichia coli* strain JM109. The pGEMT–*OvNAD1* plasmid was isolated from the bacteria for use in subsequent experiments.

#### Specificity of oligonucleotide primers and sgRNA for the *Ov*-RPA–CRISPR/Cas12a assay

To evaluate specificity, gDNAs (10 ng) of *O. viverrini*, *A. lumbricoides*, *T. trichiura*, *N. americanus*, *H. taichui*, *C. sinensis*, and *F. gigantica* were used as DNA templates for RPA. The RPA products were subsequently used as DNA templates for CRISPR/Cas12a detection. All samples were analyzed in triplicate. RPA was performed using TwistAmp™ Basic Kit (TwistDx Limited, Maidenhead, UK) following the manufacturer’s instructions. Briefly, a 50-μL reaction mixture was prepared, which contained 2.4 μL of 10 μM forward and reverse primers (each), 29.5 μL of primer-free rehydration buffer, 10 ng of DNA template adjusted to a volume of 13.2 μL in Milli-Q water, and 2.5 μL of 280 mM magnesium acetate (MgOAc). Negative (no template control) and positive (*O. viverrini* DNA) controls were incorporated into each set of reactions. The reaction mixtures were incubated at 40 °C for 20 min in a T100 thermal cycler (Bio-Rad Laboratories Inc., Hercules, CA, USA). After incubation for 4 min, the tubes were removed from the cycler, vortexed, and then spun briefly before being returned to 40 °C. Overall, 2 µL of each unpurified reaction mixture was then used as a template for CRISPR/Cas12a detection. The remaining reaction mixtures were purified using GenepHlow™PCR Cleanup Kit (Geneaid Biotech Ltd) and subjected to agarose gel electrophoresis to confirm the successful amplification of the target (281 bp).

To perform the CRISPR/Cas12a assay, 30 μL of reaction mixture containing 3 μL of NEBuffer 2.1 (100 mM NaCl, 50 mM Tris–HCl, 10 mM MgCl_2_, and 1 mM DTT; pH 7.9), 1 μL of 1 μM sgRNA, 1 μL of Lba Cas12a (NEB), and RNAse-free water were added to reach a volume of 27 μL. The reaction mixtures were then incubated at 25 °C for 10 min to generate Cas12a–sgRNA complexes using a T100 thermal cycler (Bio-Rad Laboratories). Subsequently, 1 μL of 10 μM ssDNA reporter and 2 μL of RPA product were added to the reaction mixtures. The reaction mixtures were then incubated at 37 °C for 20 min before being incubated at 65 °C for 10 min to stop the reaction. The RPA products amplified from negative and positive control DNA were used as templates for negative and positive controls in CRISPR/Cas12a detection, respectively. The fluorescence values (relative fluorescence unit, RFU) were measured at the endpoint using the CFX96 Real-Time PCR System (Bio-Rad Laboratories). Additionally, the finished reaction tubes were visually inspected using an UV transilluminator and photographed using a phone camera.

#### Detection limit of the *Ov*-RPA–CRISPR/Cas12a assay

To determine the detection limit of the *Ov*-RPA–CRISPR/Cas12a assay, tenfold serial dilutions (10 ng–10^−5^ ng) of *O. viverrini* DNA were spiked in 100 ng of copro-DNA obtained from healthy human fecal samples. The reaction mixture (50 μL) contained 2.4 μL of 10 μM forward and reverse primers (each), 29.5 μL of primer-free rehydration buffer, 100 ng of copro-DNA containing 10–10^−5^ ng of *O. viverrini* DNA adjusted to a volume of 13.2 μL in Milli-Q water, and 2.5 μL of 280 mM MgOAc. The reaction mixtures were incubated at 40 °C for 20 min. Subsequently, 2 μL of each unpurified reaction mixture was used as a template for CRISPR/Cas12a detection. To perform the CRISPR/Cas12a assay, 30 μL of a reaction mixture containing 3 μL of NEBuffer 2.1, 1 μL of 1 μM sgRNA, 1 μL of Lba Cas12a (NEB), and RNAse-free water were added to reach a volume of 27 μL. The reaction mixtures were then incubated at 25 °C for 10 min. Subsequently, 1 μL of 10 μM ssDNA reporter and 2 μL of RPA product were added to the reaction mixtures. The reaction mixtures were then incubated at 37 °C for 20 min before being incubated at 65 °C for 10 min to stop the reaction. All samples were analyzed in triplicate.

#### Determination of the optimal cutoff value

The optimal cutoff value was determined on the basis of fluorescence values obtained via the real-time PCR system. We performed the *Ov*-RPA–CRISPR/Cas12a assay of archival specimens, including *O. viverrini*-positive (*n* = 4) and *H. taichui*-positive (*n* = 10) fecal samples, which were detected via KK examination. These infected fecal specimens were additionally confirmed the species of infection by morphological identification of adult worms, which were obtained by worm expulsion. Additionally, other helminth-positive fecal samples, including *A. lumbricoides*, *T. trichiura*, hookworm, and *Taenia* sp. (each species, *n* = 3), as well as negative fecal samples (*n* = 10), were used as controls in this assay.

To extract DNA from fecal samples, approximately 200 mg of feces preserved in 80% (v/v) ethanol was transferred to a new microcentrifuge tube and washed thrice with Milli-Q sterile water to remove the ethanol. To disrupt the helminth eggs, the sediment was rapidly frozen in liquid nitrogen and lysed using TissueLyser LT (Qiagen, Hilden, Germany) with stainless-steel beads (5 mm) (Qiagen). DNA was extracted from the sediment via QIAamp® Fast DNA Stool Mini Kit (Qiagen) following the manufacturer’s instructions. DNA was eluted in 50 μL of ATE buffer. The quality and quantity of DNA were determined using NanoDrop (Thermo Fisher Scientific Inc.) and 1% agarose gel electrophoresis. All DNA samples were stored at −20 °C and then utilized for the *Ov*-RPA–CRISPR/Cas12a assay under previously described conditions. Negative copro-DNA (negative fecal sample) and pGEMT–*OvNAD1* were incorporated into each set of reactions as negative and positive controls, respectively.

Before being used in the *Ov*-RPA–CRISPR/Cas12a assay, to assess DNA quality, all DNA samples were subjected to RPA, targeting the internal control *hACTB*. The RPA conditions for *hACTB* were identical to those previously mentioned, except for the use of specific primers for *hACTB* (Additional file 1: Table S1B). The target amplicon was evaluated using 1% gel electrophoresis. To test for the presence of any inhibitory factors in the copro-DNA samples, pET20b^+^–*GsCPC2* was added to each RPA reaction mixture. Briefly, the 50-μL reaction mixture contained 2.4 μL of 10 μM forward and reverse primers (each) specific to *GsCPC2* (Additional file 1: Table S1B), 29.5 μL of primer-free rehydration buffer, 2 μL of pET20b^+^–*GsCPC2* and 2 μL of copro-DNA sample adjusted to a volume of 13.2 μL in Milli-Q water, and 2.5 μL of 280 mM MgOAc. The reaction mixtures were incubated at 40 °C for 20 min in a T100 thermal cycler (Bio-Rad Laboratories). Gel electrophoresis (1%) was employed to assess the *GsCPC2* amplicon. The samples were considered uninhibited when the DNA amplicons were 378 bp long.

A receiver operating characteristic (ROC) curve was constructed to compare the diagnostic performance of the *Ov*-RPA–CRISPR/Cas12a assay with KK method. The optimal cutoff value was calculated using easyROC v.1.3.1 (http://biosoft.erciyes.edu.tr/app/easyROC/). A *P*-value of < 0.05 was considered to indicate statistical significance. The standard error of the area under the curve (AUC) was estimated using the method described by Delong et al. [40].

### Validation of the *Ov*-RPA–CRISPR/Cas12a assay using field-collected fecal samples

#### Study area

**Fig. 1:**
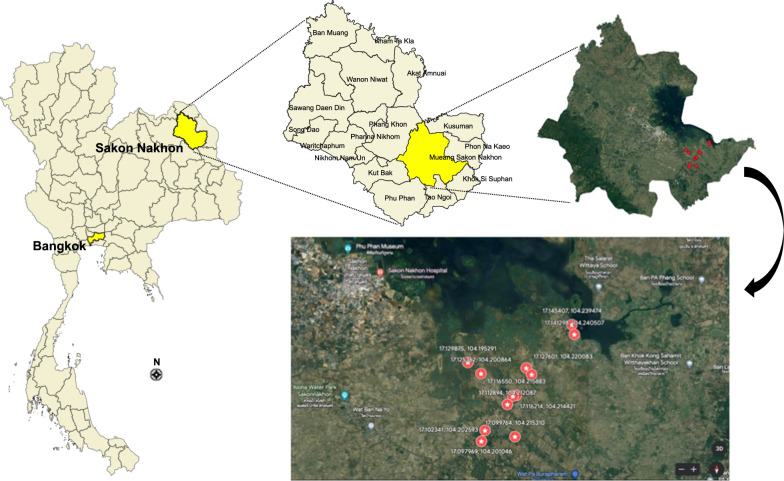
Map of Thailand showing the location of the field sites. Specific field collection sites are marked in the enlargements

The study site was located in Ban Nongplanoi, Mueang Sakon Nakhon District, Sakon Nakhon Province (Fig. 1).

#### Ethics approval and consent to participate

This study was approved by the Human Research Ethics Committee of the Faculty of Tropical Medicine, Mahidol University, Bangkok, Thailand (MUTM 2022–049-01). Written informed consent was obtained from all participants. The fecal examination results were reported to health-promoting hospitals in the targeted areas for further action by local medical doctors or health officers following treatment guidelines provided by the Department of Disease Control, Ministry of Public Health, Thailand [41].

#### Fecal sample collection

Labeled sample cups were distributed to the study participants by health volunteers. The following day, samples were either collected by health volunteers or submitted to a collection point by the participants. Along with fecal sample collection, patient information regarding age, sex, and geographical location was recorded. KK and FECT analyses were performed on-site. To perform the *Ov*-RPA–CRISPR/Cas12a assay, parallel fecal samples (approximately 200 mg) were stored in a 2-mL microcentrifuge tube containing 80% (v/v) ethanol for transport at room temperature to the Department of Helminthology, Faculty of Tropical Medicine, Bangkok.

All human fecal samples were examined using the KK method. Briefly, a stainless-steel sieve was used to press individual fecal samples, and 39.2 mg of the nonretained material was used to fill a kit template. The sample was covered with glycerin–malachite green-soaked cellophane and firmly pressed to disseminate the feces across the surface. After 30 min, the entire slide was examined using a light microscope for the presence of *O. viverrini*-like eggs and other helminths. Each fecal sample was examined in duplicate by different examiners, and the number of eggs per gram of feces (EPG) was calculated by multiplying the egg count by 25.5. All samples were anonymized.

Parallel to the KK method, FECT was performed. Briefly, 2 g of the fecal sample was mixed with 10 mL of 0.85% normal saline solution, drained through two layers of wet gauze, and collected in a 15-mL centrifuge tube. Subsequently, the sample was centrifuged at 3000 rpm for 5 min, and the supernatant was discarded. The centrifuge tube was filled with 10 mL of 10% formalin, followed by the addition of 3 mL of ethyl–acetate, shaken vigorously, and centrifuged at 3000 rpm for 5 min. After removing the top three layers, the sediment containing 1 mL of 10% formalin was retained. The final suspension was examined using a light microscope for the presence of *O. viverrini*-like eggs and other helminths.

#### *Ov*-RPA–CRISPR/Cas12a assay

All field fecal samples preserved with ethanol were washed thrice with Milli-Q sterile water and were disrupted as previously described before extracting DNA using QIAamp® Fast DNA Stool Mini Kit (Qiagen). The quality and quantity of the DNA were determined using NanoDrop (Thermo Fisher Scientific Inc.) and 1% agarose gel electrophoresis.

Overall, 100 ng of each DNA sample served as the template for RPA. The reaction mixtures and procedures for RPA and CRISPR/Cas12a detection have been described previously. Negative copro-DNA (negative fecal sample) and pGEMT-*OvNAD1* were included in each set of reactions as negative and positive controls, respectively.

The CFX96 Real-Time PCR System (Bio-Rad Laboratories, Inc.) was used to measure the fluorescence value at the endpoint for detection. The sample with an endpoint fluorescence value above the cutoff value was considered positive for *O. viverrini* infection, whereas that below the cutoff value was considered a negative result. Before the *Ov*-RPA–CRISPR/Cas12a assay, RPA was performed in all DNA samples targeting *hACTB* and *GsCPC2*. Additionally, the finished reaction tubes were visually inspected using an UV transilluminator and photographed using a phone camera and/or Gel Doc XR + Gel Documentation System (Bio-Rad Laboratories, Inc.).

#### Statistical analysis

The sensitivity and specificity of the *Ov*-RPA–CRISPR/Cas12a assay were determined via comparison with the standard methods (KK and/or FECT) using MedCalc’s diagnostic test evaluation calculator v22.005 (https://www.medcalc.org/calc/diagnostic_test.php).

A ROC curve was constructed using easyROC v1.3.1 (http://biosoft.erciyes.edu.tr/app/easyROC/) to compare the diagnostic performance of the *Ov*-RPA–CRISPR/Cas12a assay with that of the standard methods. A *P-*value of < 0.05 was considered to indicate statistical significance [40].

## Results

### Specificity of oligonucleotide primers and sgRNA for the *Ov*-RPA–CRISPR/Cas12a assay

**Fig. 2:**
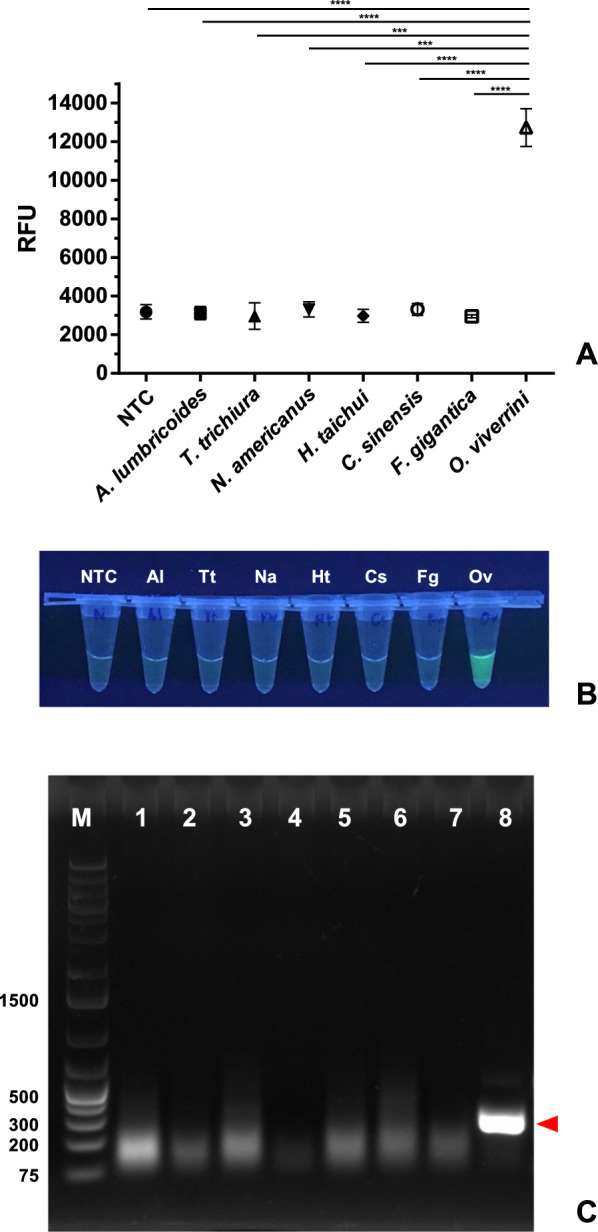
**A** Fluorescence values of the reactions containing *Opisthorchis viverrini* DNA and other helminth DNA were detected using a CFX96 Real-Time PCR System (Bio-Rad Laboratories, Inc.). Results are displayed as mean ± SD of three independent experiments. *** P < 0.001, ****P < 0.0001. **B** The image shows exposure of finished reaction tubes (*Ov*-RPA–CRISPR/Cas12a) to UV light. Only *O. viverrini* exhibited fluorescence when exposed to UV light. Tubes 1–8: nontemplate control (NTC), Al (*A. lumbricoides*)*,* Tt (*T. trichiura*)*,* Na (*N. americanus*)*,* Ht (*H. taichui*)*,* Cs (*C. sinensis*)*,* Fg (*F. gigantica*), and Ov (*O. viverrini*, positive control). All reaction tubes were visually inspected using an UV transilluminator and photographed using a phone camera. **C** The image shows agarose gel electrophoresis of the RPA products. Only the reaction containing *O. viverrini* DNA showed the target amplicon at 281 bp. Lane M: 1 kb ladder (GeneRuler 1 kb Plus DNA Ladder, Thermo Fisher Scientific Inc.). Lanes 1–8: NTC, *A. lumbricoides, T. trichiura, N. americanus, H. taichui, C. sinensis, F. gigantica*, and *O. viverrini*. One of the three replicates was subjected to 1% gel electrophoresis (0.5× TBE, 100 V, ~ 35 min) and UV light assessment

The specificity of oligonucleotide primers and sgRNA for the *Ov*-RPA–CRISPR/Cas12a assay demonstrated that the reaction containing *O. viverrini* DNA (12,742.6 ± 982.2) yielded a significantly higher fluorescent signal than that containing nontarget DNA (ranging from 2958.1 ± 79.2 to 3313.0 ± 301.3) (*P* < 0.001; Fig. 2A). Upon visual inspection, the finished reaction tube containing *O. viverrini* DNA exhibited a strong fluorescent signal when exposed to UV light, whereas the other helminth species did not exhibit such fluorescence (Fig. 2B). To confirm the specific amplification and fluorescence detection, the RPA finished reactions were purified and subjected to gel electrophoresis analysis to determine the target amplicon. The results indicated that the target RPA amplicon (281 bp) was amplified only in the reaction containing *O. viverrini* DNA (Fig. 2C).

### Detection limit of the *Ov*-RPA–CRISPR/Cas12a assay

**Fig. 3:**
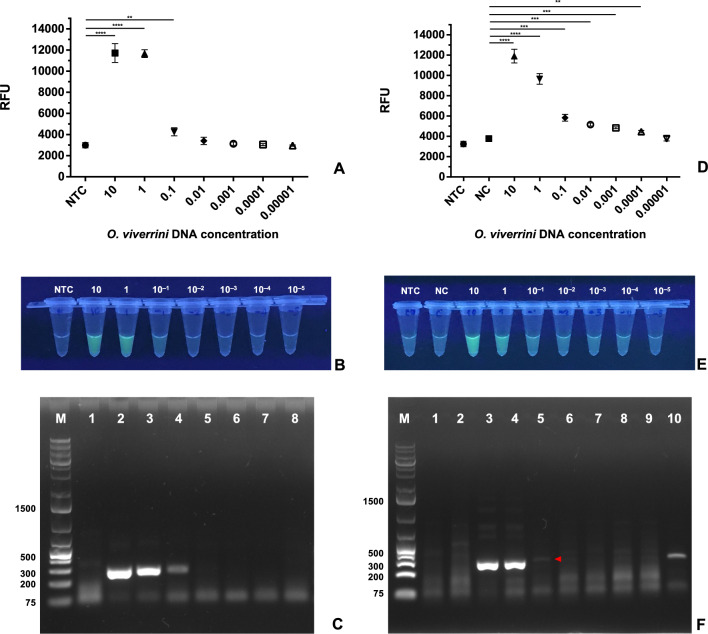
**A** Fluorescence values of tenfold serial dilutions of *Opisthorchis viverrini* DNA in Milli-Q sterile water. Results are displayed as mean ± SD of three independent experiments. ** P < 0.01, *** P < 0.001, **** P < 0.0001. **B** The image shows the exposure of finished reaction tubes (*Ov*-RPA–CRISPR/Cas12a) to UV light. Tubes 1–8: NTC, *O. viverrini* DNA at concentrations of 10, 1, 10^−1^, 10^−2^, 10^−3^, 10^−4^, and 10^−5^ ng after dilution with Milli-Q sterile water. All reaction tubes were visually inspected using an UV transilluminator and photographed using a phone camera. **C** The image shows agarose gel electrophoresis results of purified RPA products obtained from finished reactions. Lane M: 1 kb ladder. Lanes 1–8: NTC, *O. viverrini* DNA at concentrations of 10, 1, 10^−1^, 10^−2^, 10^−3^, 10^−4^, and 10^−5^ ng. The target amplicons (281 bp) were amplified at *O. viverrini* DNA concentrations of 10, 1, and 10^−1^ ng. One of the three replicates was subjected to gel electrophoresis and UV light assessment. **D** Fluorescence values of NTC, NC (*O. viverrini*-negative human copro-DNA), and *O. viverrini* DNA at concentrations of 10, 1, 10^−1^, 10^−2^, 10^−3^, 10^−4^, and 10^−5^ ng spiked in 100 ng of NC. **E** Tubes 1–9: NTC, NC, *O. viverrini* DNA at concentrations of 10, 1, 10^−1^, 10^−2^, 10^−3^, 10^−4^, and 10^−5^ ng spiked in 100 ng of NC. **F** Lane M: 1 kb ladder. Lanes 1–9: NTC, NC, *O. viverrini* DNA at concentrations of 10, 1, 10^−1^, 10^−2^, 10^−3^, 10^−4^, and 10^−5^ ng. Lane 10: 10^−1^ ng of *O. viverrini* DNA (spiked in Milli-Q sterile water). The target amplicons (281 bp) were amplified at *O. viverrini* DNA concentrations of 10, 1, and 10^−1^ ng (red arrowhead)

Tenfold serial dilutions (10 ng–10^−5^ ng) of *O. viverrini* DNA in Milli-Q sterile water were used to determine the detection limit of the *Ov*-RPA–CRISPR/Cas12a assay. The reaction containing *O. viverrini* DNA showed significantly different fluorescence values at 10 ng (11,717.0 ± 884.2; *P* < 0.0001), 1 ng (11,663.8 ± 369.3; *P* < 0.0001), and 10^−1^ ng (4265.3 ± 380.3;* P* = 0.0080) compared with NTC reactions (2991.3 ± 241.3). When reactions containing 10, 1, and 10^−1^ ng of *O. viverrini* DNA were exposed to UV light, the finished reaction tubes exhibited fluorescence (Fig. 3A, B). Although the finished reaction tubes containing *O. viverrini* DNA at a concentration of 10^−1^ appeared slightly brighter than those containing DNA at a concentration of 10^−2^, careful observation is required when conducting visual inspection. Gel electrophoresis analysis results indicated that target RPA amplicons were amplified using DNA at concentrations of 10, 1, and 10^−1^ ng, as shown in Fig. 3C.

When *O. viverrini* DNA at various concentrations (10, 1, 10^−1^, 10^−2^, 10^−3^, 10^−4^, and 10^−5^ ng) was spiked into 100 ng of *O. viverrini*-negative human copro-DNA, the reactions containing *O. viverrini* DNA exhibited significantly different fluorescence values at 10 ng (11,909.7 ± 676.5; *P* < 0.0001), 1 ng (9654.0 ± 527.2; *P* < 0.0001), 10^−1^ ng (5835.0 ± 338.6; *P* = 0.0007), 10^−2^ ng (5161.8 ± 146.4; *P* = 0.0005), 10^−3^ ng (4833.6 ± 93.0; *P* = 0.0008), and 10^−4^ ng (4462.3 ± 136.0; *P* = 0.0063) compared with those containing *O. viverrini*-negative human copro-DNA (NC) (3785.5 ± 177.3; healthy fecal samples; Fig. 3D). Upon visual inspection, the finished reaction tubes containing 10 and 1 ng of *O. viverrini* DNA exhibited obvious fluorescence when exposed to UV light (Fig. 3E). However, the finished reaction tubes containing *O. viverrini* DNA at concentrations of 10^−1^ and lower did not show significant visual differences. Regarding gel electrophoresis analysis, the RPA amplicons were detected at a DNA concentration as low as 10^−1^ ng (Fig. 3F).

On the basis of visual inspection, the *Ov*-RPA–CRISPR/Cas12a assay showed a sensitivity of as low as 1 ng for detecting *O. viverrini* DNA. On the basis of statistical analysis of the fluorescence values, the *Ov*-RPA–CRISPR/Cas12a assay had a sensitivity of as low as 10^−4^ ng for detecting *O. viverrini* DNA. However, gel electrophoresis could only detect *O. viverrini* DNA at a concentration of 10^−1^ ng, indicating the production of fluorescence background noise at DNA concentrations of 10^−2^ ng and lower. Therefore, the detection limit was determined to be between 1 and 10^−1^ ng of DNA, on the basis of visual inspection and fluorescence values.

### Determination of the optimal cutoff value using ROC curve analysis

To determine the optimal cutoff value, the *Ov*-RPA–CRISPR/Cas12a assay was conducted using 26 positive and 10 negative fecal samples. The ROC curve was assessed using the fluorescence values obtained from the real-time PCR system. The results revealed that 36 fecal samples showed fluorescence values ranging from 3470.2 to 10,771.8 (Additional file 3: Table S2). The fluorescence values of the four samples that were positive for *O. viverrini* infection ranged from 4620.7 to 10,771.8 (sample IDs: Ov01–Ov04) (Additional file 3: Table S2). Upon visual inspection, compared with negative and positive controls, finished reaction tubes 1 (sample ID: Ov01), 3 (sample ID: Ov03), and 4 (sample ID: Ov04) exhibited obvious fluorescence under UV light, whereas finished reaction tube 2 (sample ID: Ov02) exhibited weak fluorescence (Additional file 4: Fig. S2A). On the basis of gel electrophoresis analysis, sample tubes 1, 2, 3, and 4 contained RPA amplicons compared with positive controls (Additional file 4: Fig. S2B). The results revealed that *O. viverrini* infection was detected in all four samples.

In the *Ov*-RPA–CRISPR/Cas12a assay using 10 *H. taichui*-positive fecal samples (sample IDs: Ht01–Ht10), three samples showed fluorescence values above the cutoff value: 7341.9 (sample ID: Ht01), 4681.1 (sample ID: Ht04), and 6358.3 (sample ID: Ht07) (Additional file 3: Table S2). Upon visual inspection, two of the three samples, i.e., finished reaction tubes 1 (sample ID: Ht01) and 7 (sample ID: Ht07), showed fluorescence when exposed to UV light (Additional file 4: Fig. S2C). Conversely, the finished reaction tube 4 (sample ID: Ht04) produced fluorescence that was indistinguishable from the fluorescence background noise. When assessed via gel electrophoresis, only sample IDs Ht01 and Ht07 showed target RPA amplicons as faint bands (Additional file 4: Fig. S2D). The inverted gel electrophoresis image is presented in Additional file 5: Fig. S3, indicating the presence of *O. viverrini* infection.

In the *Ov*-RPA–CRISPR/Cas12a assay using 10 negative fecal samples (sample IDs: N01–N10) and 12 samples positive for other helminths (sample IDs: Al01–Al03, Tt01–Tt03, Hw01–Hw03, and Tae01–Tae03), the negative fecal samples generated fluorescence values ranging from 3470.2 to 4173.9, whereas those positive for other helminths generated fluorescence values ranging from 3539.8 to 3754.1 (Additional file 3: Table S2). Upon visual inspection, none of the finished reaction tubes showed fluorescence under UV light (Additional file 4: Fig. S2E, G). Furthermore, none of the samples contained target RPA amplicons, indicating the absence of *O. viverrini* infection (Additional file 4: Fig. S2F, H).

To determine the optimal cutoff value via ROC curve analysis, we used the fluorescence values obtained from the *Ov*-RPA–CRISPR/Cas12a assay of all samples. The results revealed that the AUC was 0.97 (95% CI 0.85–1.00; *P* < 0.0001), indicating an excellent capacity to distinguish positive and negative samples. The optimal cutoff value was determined to be 4620.7 (Additional file 6: Table S3). Cutoff plots are shown in Additional file 7: Fig. S4.

### Diagnostic performance of the *Ov*-RPA–CRISPR/Cas12a assay using field-collected fecal samples

#### Fecal examination using the KK and FECT methods

A total of 172 fecal samples were collected from all study participants residing in Ban Nongplanoi, Mueang Sakon Nakhon District, Sakon Nakhon Province. KK and FECT methods were used to analyze all fecal samples for helminthic infections.

According to the KK and FECT results, 29 (16.9%) and 35 (20.3%) fecal samples were determined to be positive for *O. viverrini*-like eggs, respectively, whereas 143 (83.1%) and 137 (79.7%) fecal samples were considered negative (Table 1). Using the KK method, 29 fecal samples showed positivity for *O. viverrini*-like eggs, of which 27 (15.7%) samples showed a single infection with *O. viverrini*-like eggs. Two additional fecal samples demonstrated coinfection with hookworms (1, 0.6%) and *Taenia* sp. (1, 0.6%). Moreover, three fecal samples showed a single infection with hookworms.

**Table 1:** Number of fecal samples with *Opisthorchis viverrini*-like eggs and other helminth infections detected using KK and FECT methods

Results	Helminth species	KK method (%)	FECT method (%)
Positive samples			
Single infection	*O. viverrini*-like egg	27 (15.7)	33 (19.2)
	Hookworm	3 (1.7)	0 (0.0)
	*S. stercoralis*	0 (0.0)	2 (1.2)
Coinfection	*O. viverrini*-like egg and hookworm	1 (0.6)	0 (0.0)
	*O. viverrini*-like egg and *Taenia* sp.	1 (0.6)	2 (1.2)
Total positive *O. viverrini*-like egg samples		29 (16.9)	35 (20.3)
Total negative *O. viverrini*-like egg samples		143 (83.1)	137 (79.7)
Total positive samples		32 (18.6)	37 (21.5)
Total negative samples		140 (81.4)	135 (78.5)
Total samples		172 (100.0)	172 (100.0)

*KK* Kato–Katz method, *FECT* formalin ethyl–acetate concentration technique

The FECT method revealed that 35 (20.3%) fecal samples were positive for *O. viverrini*-like eggs, of which 33 (19.2%) showed a single infection with *O. viverrini*-like eggs and two samples demonstrated coinfection with *Taenia* sp. (2, 1.2%). The FECT method revealed that two fecal samples tested positive for *Strongyloides stercoralis.*

#### *Ov*-RPA–CRISPR/Cas12a assay

To assess the quality of copro-DNA, all DNA samples were subjected to RPA targeting the internal control *hACTB* and *GsCPC2*. Of 172 DNA samples, 121 showed positive amplification of *hACTB* (221 bp) and *GsCPC2* (378 bp) amplicons (Additional file 8: Table S4 and Additional file 9: Fig. S5). Therefore, the *Ov*-RPA–CRISPR/Cas12a assay was performed using 121 copro-DNA samples. On the basis of the determined cutoff value (fluorescence value ≥ 4620.7) using a real-time PCR system, 28 samples were determined to be positive for *O. viverrini* infection, whereas 93 samples were deemed negative (Additional file 8: Table S4).

A comparison of the results of the *Ov*-RPA–CRISPR/Cas12a assay, based on fluorescence values, with those of KK and/or FECT methods, revealed 18 true positives, 82 true negatives, 10 false positives, and 11 false negatives (Additional file 8: Table S4). These results indicate that the *Ov*-RPA–CRISPR/Cas12a assay exhibits sensitivity and specificity of 62.1% and 89.1%, respectively (Table 2). On the basis of a 50% prevalence of *O. viverrini*, the calculated positive predictive value (PPV), negative predictive value (NPV), positive likelihood ratio (LR+), and negative LR (LR−) were 85.1%, 70.2%, 5.7, and 0.4, respectively.

**Table 2:** Diagnostic performance of the *Ov*-RPA–CRISPR/Cas12a assay based on the fluorescence values for detecting *O. viverrini* infection in field-collected fecal samples (*n* = 121) compared with standard methods (KK and/or FECT methods)

Diagnostic performance of the *Ov*-RPA–CRISPR/Cas12a assay	Value	95% CI
Sensitivity	62.1%	42.3–79.3%
Specificity	89.1%	81.0–94.7%
Positive likelihood ratio (LR+)	5.7	3.0–11.0
Negative likelihood ratio (LR−)	0.4	0.3–0.7
Positive predictive value (PPV)	85.1%	74.9–91.6%
Negative predictive value (NPV)	70.2%	59.5–79.0%
Accuracy	75.6%	67.0–83.0%

*LR* likelihood ratio, *PPV* positive predictive value, *NPV* negative predictive value, *KK* Kato–Katz method, *FECT* formalin ethyl–acetate concentration technique. LRs were estimated using a 50% prevalence rate of *O. viverrini*

**Fig. 4:**
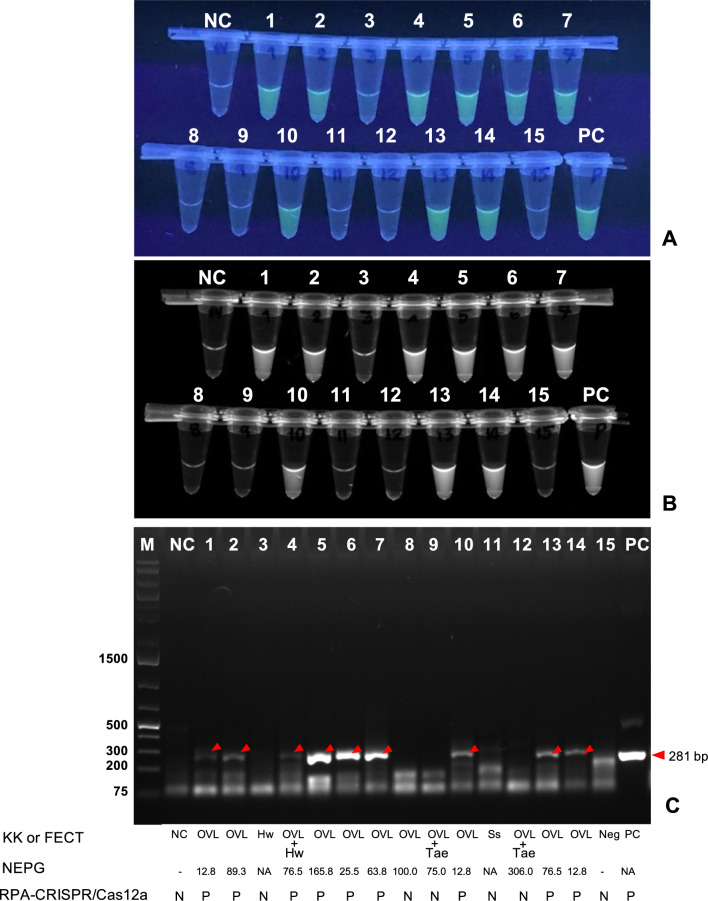
**A** Representation of the results of finished reaction tubes (*Ov*-RPA–CRISPR/Cas12a) when exposed to UV light. On the basis of KK and/or FECT methods, tube NC contained *O. viverrini*-negative human copro-DNA, and tubes 1, 2, 5, 6, 7, 8, 10, 13, and 14 were positive for *O. viverrini*-like eggs. Tube 4 showed positive results for the coinfection of *O. viverrini*-like eggs and hookworms. Tubes 9 and 12 exhibited positivity for the coinfection of *O. viverrini*-like eggs and *Taenia* sp., respectively. Tubes 3 and 11 were positive for hookworms and *S. stercoralis*, respectively. Tube 15 represented a negative fecal sample, whereas tube PC served as a positive control with a plasmid containing the *O. viverrini* *NAD1* gene. All reaction tubes were visually inspected using an UV transilluminator and photographed using a phone camera. **B** The image was recorded using the Gel Doc XR + Gel Documentation System. The tube’s label is based on image A. **C** The image shows agarose gel electrophoresis results of RPA products, with the red arrow indicating the target amplicon (281 bp). The lane labels align with images A and B. Additional details, including the results of KK and/or FECT methods, number of eggs per gram (NEPG), and *Ov*-RPA–CRISPR/Cas12a assay, are shown below the image. Lane M: 1 kb ladder. 1% gel electrophoresis (0.5× TBE, 100 V, ~ 35 min)

Figure 4A–C shows the results of the *Ov*-RPA–CRISPR/Cas12a assay obtained from finished reaction tubes exposed to UV light and RPA products from gel electrophoresis. On the basis of KK and/or FECT methods, 12 samples were considered positive for *O. viverrini*-like eggs. Nine of these samples generated fluorescent signals above the cutoff value (fluorescence value ≥ 4620.7) when assessed using a real-time PCR system (Additional file 8: Table S4), indicating a positive result for *O. viverrini* infection. Samples of other infected helminths, such as hookworms (Fig. 4A, B tube 3 and Fig. 4C lane 3) and *S. stercoralis* (Fig. 4A, B tube 11 and Fig. 4C lane 11), displayed fluorescence signals below the cutoff value, indicating the absence of *O. viverrini*. Notably, on the basis of fluorescence values, three samples—one with a single *O. viverrini*-like infection (Fig. 4A, B tube 8 and Fig. 4C lane 8) and two with coinfections of *O. viverrini* and *Taenia* sp. (Fig. 4A, B tubes 9, 12 and Fig 4C lanes 9, 12), exhibited false-negative results.

Although the assessments were conducted on the basis of fluorescence values using a real-time PCR system, an UV transilluminator was used to assess the reaction tubes. This is because the tubes displayed a fluorescence signal when exposed to UV light (Fig. 4A). Figure 4B displays the results of finished reaction tubes according to Fig. 4A, recorded using the Gel Doc XR + Gel Documentation System. Figure 4C presents the DNA amplicons generated via RPA according to Fig. 4A, B, indicating that the target amplicon (red arrowhead) matched the positive finished reaction.

### ROC curve analysis

**Fig. 5:**
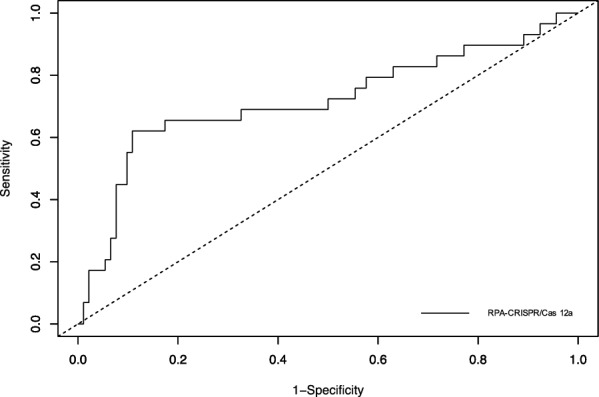
Receiver operating characteristic (ROC) curve analysis of the comparison between *Ov*-RPA–CRISPR/Cas12a assay and the standard methods (KK and/or FECT) for detecting *O. viverrini* infection in field-collected fecal samples (*n* = 121)

A ROC curve was constructed using the fluorescence values obtained from 121 field-collected fecal samples. As shown in Fig. 5, the ROC curve analysis of the comparison of *Ov*-RPA–CRISPR/Cas12a assay and KK and/or FECT methods revealed an AUC of 0.72 (95% CI 0.63–0.80) (*P* = 0.00058), suggesting an appropriate discriminating power of the assay. Method performance, ROC statistics, and ROC coordinates are shown in Additional file 10: Table S5 and Additional file 11: Fig. S6.

## Discussion

Although several molecular-based methods have demonstrated high sensitivity and specificity for detecting *O. viverrini* infection [13–22], RPA–CRISPR/Cas12a is a novel technique that can detect several pathogens with high sensitivity and specificity [24–31]. However, RPA–CRISPR/Cas12a has not yet been established for the diagnosis of *O. viverrini* infection. Therefore, this study aimed to use this technology to develop a diagnostic method for *O. viverrini* infection in fecal samples and evaluate its efficacy. Additionally, its application revealed the feasibility of a field-deployable approach in the field setting [34, 35].

In addition to the development of a novel, accurate diagnostic method, the copro-DNA extraction process presents a challenging aspect that needs to be validated. Fecal samples contain many inhibitory substances that are unfavorable for molecular assays, necessitating the use of high-efficiency DNA extraction kits, which are expensive and involve multiple isolation steps. Moreover, disrupting the parasiteʼs outer eggshell often requires additional physical disruption [42]. On the basis of the study protocol, *O. viverrini* eggs were disrupted using specific methods and instruments, including liquid nitrogen, stainless-steel beads, and TissueLyser LT machine, to facilitate DNA extraction. DNA extraction remains one of the major challenges in developing CRISPR/Cas as a point-of-care (POC) diagnostic tool. Although several methods, including freeze–thaw cycles, heating or boiling, shaking with glass beads, and digestion with proteinase K, have been proposed to disrupt the outer eggshell [43, 44], further research is warranted to determine the most effective approach for extracting *O. viverrini* copro-DNA in the field.

To ensure the reliability and accuracy of the study findings, all DNA samples from feces were examined for the presence of internal control DNA and inhibitory substances before conducting *Ov*-RPA–CRISPR/Cas12a assay. Additionally, copro-DNA samples of *O. viverrini*- and *H. taichui*-positive fecal samples, from which adult worms were used to confirm species by morphological identification, were used to establish the optimal cutoff value for fluorescence detection. To validate the outcomes, all reactions that yielded a positive result in the *Ov*-RPA–CRISPR/Cas12a assay were purified, and gel electrophoresis was used to confirm the presence of the *OvNAD1* target amplicon.

On the basis of the fluorescence value, the *Ov*-RPA–CRISPR/Cas12a assay indicated a cutoff value of 4620.7 for *O. viverrini* positivity. Consequently, 3 of 10 *H. taichui*-positive samples showed positivity for *O. viverrini* infection, as evidenced by fluorescence values that surpassed the above-mentioned cutoff value. This suggests that the cause could be coinfection with both species. On the basis of epidemiology, coinfection is common because both opisthorchiasis and heterophyidiasis often occur in the same endemic area [11, 12, 45]. Regarding the three samples that tested positive in the *Ov*-RPA–CRISPR/Cas12a assay but negative for worm expulsion, it is possible that a mild *O. viverrini* infection resulted in *O. viverrini* worms being undetected in *H. taichui-*positive samples. This result demonstrates the effectiveness of the developed method. This study provides excellent representative samples for determining the cutoff value in real-world setting.

Although the *Ov*-RPA–CRISPR/Cas12a assay developed in this study demonstrated high specificity, it exhibited limited sensitivity, detecting 11 of 29 fecal samples as false negatives. The NEPG in nine of these samples was > 50, indicating values higher than the detection limit of the method. Amplifying the internal control and known spiked DNA allows us to rule out interference from inhibitory substances in the feces and poor DNA extraction quality. Although the egg morphology observed in these samples resembles that of *O. viverrini* (Additional file 12: Fig. S7), definitive confirmation of *O. viverrini* eggs is challenging due to their shared characteristics with *Haplorchis* eggs [11, 12, 46]. To definitively determine the species, worm expulsion must be performed. Egg disruption during DNA extraction may have prevented the detection of *O. viverrini* infection, limiting the full DNA yield. Additionally, a small amount of fecal material obtained from a sample with mild infection for DNA isolation might not contain *O. viverrini* eggs, leading to a false-negative result for the *Ov*-RPA–CRISPR/Cas12a assay. However, the discrepancies between these approaches warrant further verification using additional methods.

It is well known that the *trans*-cleavage activity of the CRISPR/Cas12a system starts when the Cas12a/sgRNA complex binds to target sequences [39]. Consequently, our developed assay can initiate *trans*-cleavage activity and successfully detect the target. Upon visual inspection using an UV transilluminator, the positive signal from the *Ov*-RPA–CRISPR/Cas12a assay was visible at a minimal DNA concentration of 1 ng. On the basis of our findings, at an *O. viverrini* DNA concentration of 10^−1^ ng, the assay generated a weak fluorescent signal, which was challenging to distinguish from background noise during visual inspection. A limitation of this study was encountered when *O. viverrini* DNA concentration was as low as 10^−1^ ng. Visual interpretation without gel electrophoresis could not distinguish the fluorescence of the positive finished reaction from the background noise. Therefore, a real-time PCR system was used to determine the fluorescence value to confirm the detection results. This system demonstrated improved sensitivity to detect *O. viverrini* DNA at a minimum concentration of 10^−1^ ng. Consequently, we could overcome the limitations of visual interpretation.

The finished reaction of *O. viverrini* DNA (10^−2^ ng and lower) with *O. viverrini*-negative human copro-DNA (100 ng) generated fluorescence background noise when compared with that of *O. viverrini* DNA without *O. viverrini*-negative human copro-DNA (in Milli-Q water), indicating that *O. viverrini*-negative human copro-DNA influences the generation of the fluorescence background noise. Nonspecific amplification is prone to occur during RPA, particularly in samples with high background DNA concentrations [32]. Consequently, unexpected RPA products in the CRISPR/Cas12a reaction may affect the fluorescence background noise. However, a previous study found that adding betaine to the RPA reaction could significantly increase its specificity and yield [47]. Moreover, another study reported that the fluorescence background noise can be minimized by shortening the ssDNA reporter sequence. Under identical conditions, the study demonstrated that a 5-nt ssDNA reporter exhibited higher sensitivity and lower background than a longer ssDNA reporter [48]. Although current fluorescent reporters still rely on original pioneering studies [39, 49, 50], future research should explore this approach to improve the signal-to-background ratio of our currently developed assay. Although the fluorescent signal can be visually assessed, fluorescence values and gel electrophoresis were used to determine the results, allowing a positive fluorescence to be distinguished from background noise.

In the future, we want to test a portable fluorescence reader because the real-time PCR system used in this study is unsuitable for a POC setting. Currently, several companies and research groups have developed prototype portable fluorescent signal readouts for RPA–CRISPR/Cas detection [51–54]. In addition to developing portable fluorescent signal readouts, several studies have integrated lateral flow strips into the RPA–CRISPR/Cas detection system, recommending its dependability and use in field locations with limited resources [55–57].

In this study, we used UV and blue light LED transilluminators to visualize the results. However, when the finished reactions were observed under blue light LED transilluminators, an excessive amount of brightness was produced, showing a strong fluorescence background when compared with UV transilluminator, making it incredibly difficult to interpret the result and potentially resulting in misdiagnosis. Consequently, an UV transilluminator was employed for visual inspection. However, due to the generation of fluorescence background noise, it was challenging to visually distinguish the positive fluorescent signal from the fluorescence background noise when the DNA concentration was as low as 10^−1^ ng. Interpretation based solely on visual inspection could lead to a misdiagnosis. Therefore, the fluorescence values generated by the real-time PCR system and gel electrophoresis were used to determine the results. Nevertheless, to assess the positive fluorescent signal by visual inspection alone, a reaction tube containing 1 ng of *O. viverrini* DNA must be included in the experiment. This limitation represents an aspect that requires improvement in subsequent studies.

The DNA extraction process restricts the application of *Ov*-RPA–CRISPR/Cas12a in field investigations. Therefore, it is imperative to develop a simple method to obtain DNA from *O. viverrini* eggs, such as NaOH-based DNA extraction [58], which could be applied in field settings. Additionally, implementing the *Ov*-RPA–CRISPR/Cas12a assay using lateral flow strips may facilitate POC diagnosis.

## Conclusions

The detection of *O. viverrini* infection in field-collected feces is possible using the *Ov*-RPA–CRISPR/Cas12a assay developed in this study. This assay offers a viable alternative method to confirm *O. viverrini* infection with high specificity. The *Ov*-RPA–CRISPR/Cas12 assay developed in this study will be the initial step toward developing a POC diagnostic tool. However, this assay requires a field-deployable DNA extraction method to be suitable for POC diagnosis.

### Supplementary Information

**Additional file 1: Table S1. A** Nucleotide sequences used to create oligonucleotide primers and single-guide RNA (sgRNA). **B** Oligonucleotide primers and probes used in *Ov*-RPA–CRISPR/Cas12a assay.

**Additional file 2: Figure S1.** Multiple sequence alignment shows the locations of the primer pair and sgRNA.

**Additional file 3: Table S2.** Fluorescence values determined via the *Ov*-RPA–CRISPR/Cas12a assay.

**Additional file 4: Figure S2.** Finished *Ov*-RPA–CRISPR/Cas12a reaction tubes exposed to UV light.

**Additional file 5: Figure S3.** Inverted gel electrophoresis image of Figure S2D.

**Additional file 6: Table S3.** ROC curve analysis results to obtain the optimal cutoff value.

**Additional file 7: Figure S4.** Cutoff plots obtained from ROC curve analysis comparing the *Ov*-RPA–CRISPR/Cas12a assay and KK method for detecting *O. viverrini* infection in fecal samples.

**Additional file 8: Table S4.** Results of the examination of *Opisthorchis viverrini* infection in field-collected fecal samples using the KK, FECT, and *Ov*-RPA–CRISPR/Cas12a methods.

**Additional file 9: Figure S5.** Examples of RPA amplicons targeting human actin beta gene (*hACTB*) and pET20b^+^–*GsCPC2* (*GsCPC2*).

**Additional file 10: Table S5.** ROC curve analysis results comparing the *Ov*-RPA–CRISPR/Cas12a assay and standard methods (KK and/or FECT) for detecting *O. viverrini* infection in fecal samples.

**Additional file 11: Figure S6.** Cutoff plots obtained from ROC curve analysis comparing *Ov*-RPA–CRISPR/Cas12a assay and standard methods (KK and/or FECT) for detecting *O. viverrini* infection in fecal samples.

**Additional file 12:** **Figure S7.** Examples of *O. viverrini*-like egg morphology.

## Data Availability

All data supporting the findings of this study are available within the paper and its Supplementary Information.
