# Supplementary material for: Ov-RPA–CRISPR/Cas12a assay for the detection of Opisthorchis viverrini infection in field-collected human feces
Source: Parasit Vectors. 2024 Feb 21;17:80. doi: 10.1186/s13071-024-06134-7 (PMC10882828; doi:10.1186/s13071-024-06134-7)
Supplement: Supplementary file 1 — Additional file 1: Table S1. A Nucleotide sequences used to create oligonucleotide primers and single-guide RNA (sgRNA). B Oligonucleotide primers and probes used in Ov-RPA–CRISPR/Cas12a assay. [file 13071_2024_6134_MOESM1_ESM.docx]

**Additional File 1: Table S1. A.** Nucleotide sequences used to create oligonucleotide primers and single-guide RNA (sgRNA).

| **No.** | **GenBank**  **Accession number** | **Species** |
| --- | --- | --- |
| 1 | DQ882172.1 | *Opisthorchis viverrini* strain BD NADH dehydrogenase subunit 1 gene (Vietnam) |
| 2 | DQ882174.1 | *Opisthorchis viverrini* strain DL3 NADH dehydrogenase subunit 1 gene (Vietnam) |
| 3 | DQ882175.1 | *Opisthorchis viverrini* strain OvL NADH dehydrogenase subunit 1 gene (Vietnam) |
| 4 | GQ401025.1 | *Opisthorchis viverrini* isolate VT/1 NADH dehydrogenase subunit 1 (nad1) gene (Thailand, TropMed) |
| 5 | GQ401064.1 | *Opisthorchis viverrini* isolate CP/1 NADH dehydrogenase subunit 1 (nad1) gene (Thailand, TropMed) |
| 6 | GQ401046.1 | *Opisthorchis viverrini* isolate KM/7 NADH dehydrogenase subunit 1 (nad1) gene (Thailand, TropMed) |
| 7 | GQ401060.1 | *Opisthorchis viverrini* isolate SV/12 NADH dehydrogenase subunit 1 (nad1) gene (Thailand, TropMed) |
| 8 | GQ401082.1 | *Opisthorchis viverrini* isolate KD/1 NADH dehydrogenase subunit 1 (nad1) gene (Thailand, TropMed) |
| 9 | GQ401096.1 | *Opisthorchis viverrini* isolate SK/1 NADH dehydrogenase subunit 1 (nad1) gene (Thailand, TropMed) |

**Additional File 1: Table S1. A (Cont.).** Nucleotide sequences used to create oligonucleotide primers and single-guide RNA (sgRNA).

| **No.** | **GenBank**  **Accession number** | **Species** |
| --- | --- | --- |
| 10 | EU022343.1 | *Opisthorchis viverrini* isolate KS NADH dehydrogenase subunit 1 (nad1) gene (Thailand, Khon Kaen) |
| 11 | EU022346.1 | *Opisthorchis viverrini* isolate LP NADH dehydrogenase subunit 1 (nad1) gene (Thailand, Khon Kaen) |
| 12 | EU022348.1 | *Opisthorchis viverrini* isolate SK NADH dehydrogenase subunit 1 (nad1) gene (Thailand, Khon Kaen) |
| 13 | EU022350.1 | *Opisthorchis viverrini* isolate MS NADH dehydrogenase subunit 1 (nad1) gene (Thailand, Khon Kaen) |
| 14 | FJ381664.2 | *Clonorchis sinensis*FJ381664.2:5251-6153 *Clonorchis sinensis* mitochondrion, ND1 |
| 15 | EU921260.2 | *Opisthorchis felineus*  EU921260.2:5253-6155 *Opisthorchis felineus* mitochondrion, ND1 |
| 16 | AF216697.1 | *Fasciola hepatica*  AF216697.1:5176-6078 *Fasciola hepatica* mitochondrion, ND1 |

**Additional File 1: Table S1. A (Cont.).** Nucleotide sequences used to create oligonucleotide primers and single-guide RNA (sgRNA).

| **No.** | **GenBank**  **Accession number** | **Species** |
| --- | --- | --- |
| 17 | MH621335.1 | *Fasciola gigantica*  MH621335.1:5171-6073 *Fasciola gigantica* mitochondrion, ND1 |
| 18 | NC_022433.1 | *Haplorchis taichui*  NC_022433.1:5161-6066 *Haplorchis taichui* mitochondrion, ND1 |
| 19 | KX169163.1 | *Fasciolopsis buski*  KX169163.1:5213-6115 *Fasciolopsis buski* mitochondrion, ND1 |
| 20 | AF026279.1 | *Echinostoma* sp. PCerc-4 ND1  AF026279.1 Echinostoma sp. PCerc-4 nicotinamide adenine dinucleotide dehydrogenase subunit 1 (ND1) gene |
| 21 | MT299949.1 | *Haplorchis pumilio* ND1  MT299949.1 *Haplorchis pumilio* nicotinamide dehydrogenase subunit 1 (ND1) gene |
| 22 | JF739555.1 | *Opisthorchis viverrini* mitochondrion, partial genome  JF739555.1:5246-6148 *Opisthorchis viverrini* mitochondrion (China) |

**Additional File 1: Table S1. A (Cont.).** Nucleotide sequences used to create oligonucleotide primers and single-guide RNA (sgRNA).

| **No.** | **GenBank**  **Accession number** | **Species** |
| --- | --- | --- |
| 23 | GQ401098.1 | *Opisthorchis viverrini* nd1  *Opisthorchis viverrini* isolate SK/3 NADH dehydrogenase subunit 1 (nad1) gene (Thailand, TropMed) |
| 24 | GQ401042.1 | *Opisthorchis viverrini* (nad1) gene  *Opisthorchis viverrini* isolate KM/3 NADH dehydrogenase subunit 1 (nad1) gene (Thailand, TropMed) |
| 25 | NG_007992.1 | *Homo sapiens* actin beta (ACTB), refSeqGene (LRG_132) on chromosome 7 |

**Additional File 1: Table S1. B.** Oligonucleotide primers and probes used in *Ov*-RPA–CRISPR/Cas12a assay.

| **No.** | **Primer/**  **Probe** | **Sequence**  **(5’ - 3’)** | **RPA product Size (bp)** |
| --- | --- | --- | --- |
| 1 | *Ov*_NAD1 | Forward: TGTATGTCCTATGATGGGGGTTGCAATTCGGTG | 281 |
|  |  | Reverse: CACACGACATAACACACGGGTACAACTAAAC |  |
| 2 | hACTB | Forward: TTTTAATAGTCATTCCAAATATGAGATGCGTT | 221 |
|  |  | Reverse: CGAAGGCTCATCATTCAAAATAAAACAAAAT |  |
| 3 | GsCPC2 | Forward: CGCGGATCCGCAAGATTTCTTTATTGC CGGACAG | 378 |
|  |  | Reverse: CCGCTCGAGTGAAAGTATGAATGTAAC ATGGGTAG |  |
| 4 | sgRNA | UGCUUUGGUGUGCGGAAAUUAUG |  |
| 5 | ssDNA | -6-FAM-TTTTTTTTTTTT-BHQ1 |  |
