# Supplementary material for: Ov-RPA–CRISPR/Cas12a assay for the detection of Opisthorchis viverrini infection in field-collected human feces
Source: Parasit Vectors. 2024 Feb 21;17:80. doi: 10.1186/s13071-024-06134-7 (PMC10882828; doi:10.1186/s13071-024-06134-7)
Supplement: Supplementary file 2 — Additional file 2: Figure S1. Multiple sequence alignment shows the locations of the primer pair and sgRNA. [file 13071_2024_6134_MOESM2_ESM.docx]

**Additional File 2: Figure S1.** Multiple sequence alignment shows the locations of the primer pair and sgRNA.

**Additional File 2: Figure S1 (Cont.).** Multiple sequence alignment shows the locations of the primer pair and sgRNA.
