# Supplementary material for: Ov-RPA–CRISPR/Cas12a assay for the detection of Opisthorchis viverrini infection in field-collected human feces
Source: Parasit Vectors. 2024 Feb 21;17:80. doi: 10.1186/s13071-024-06134-7 (PMC10882828; doi:10.1186/s13071-024-06134-7)
Supplement: Supplementary file 4 — Additional file 4: Figure S2. Finished Ov-RPA–CRISPR/Cas12a reaction tubes exposed to UV light. [file 13071_2024_6134_MOESM4_ESM.pptx]

## Slide 1
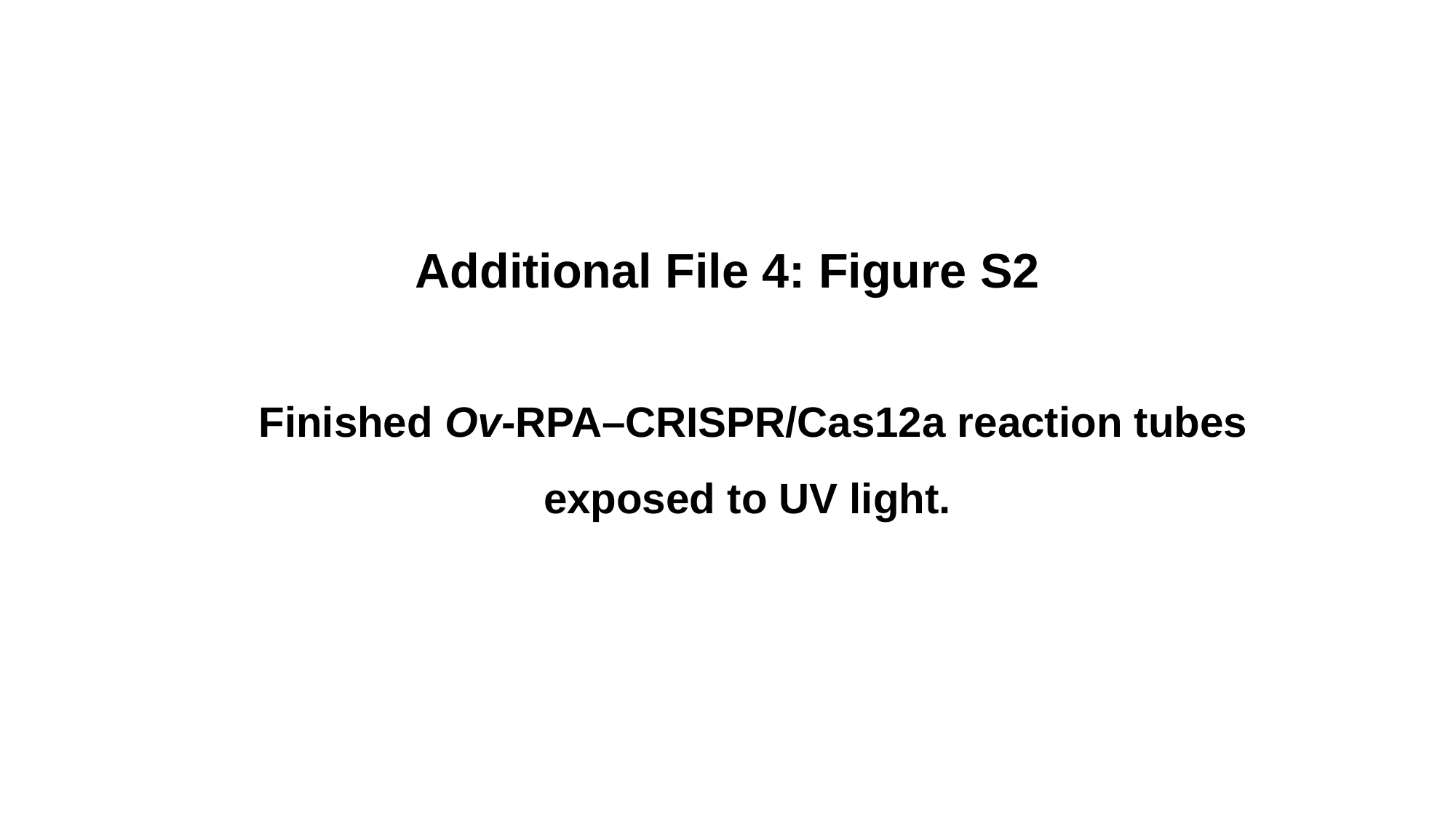

Additional File 4: Figure S2
Finished Ov-RPA–CRISPR/Cas12a reaction tubes exposed to UV light.

## Slide 2
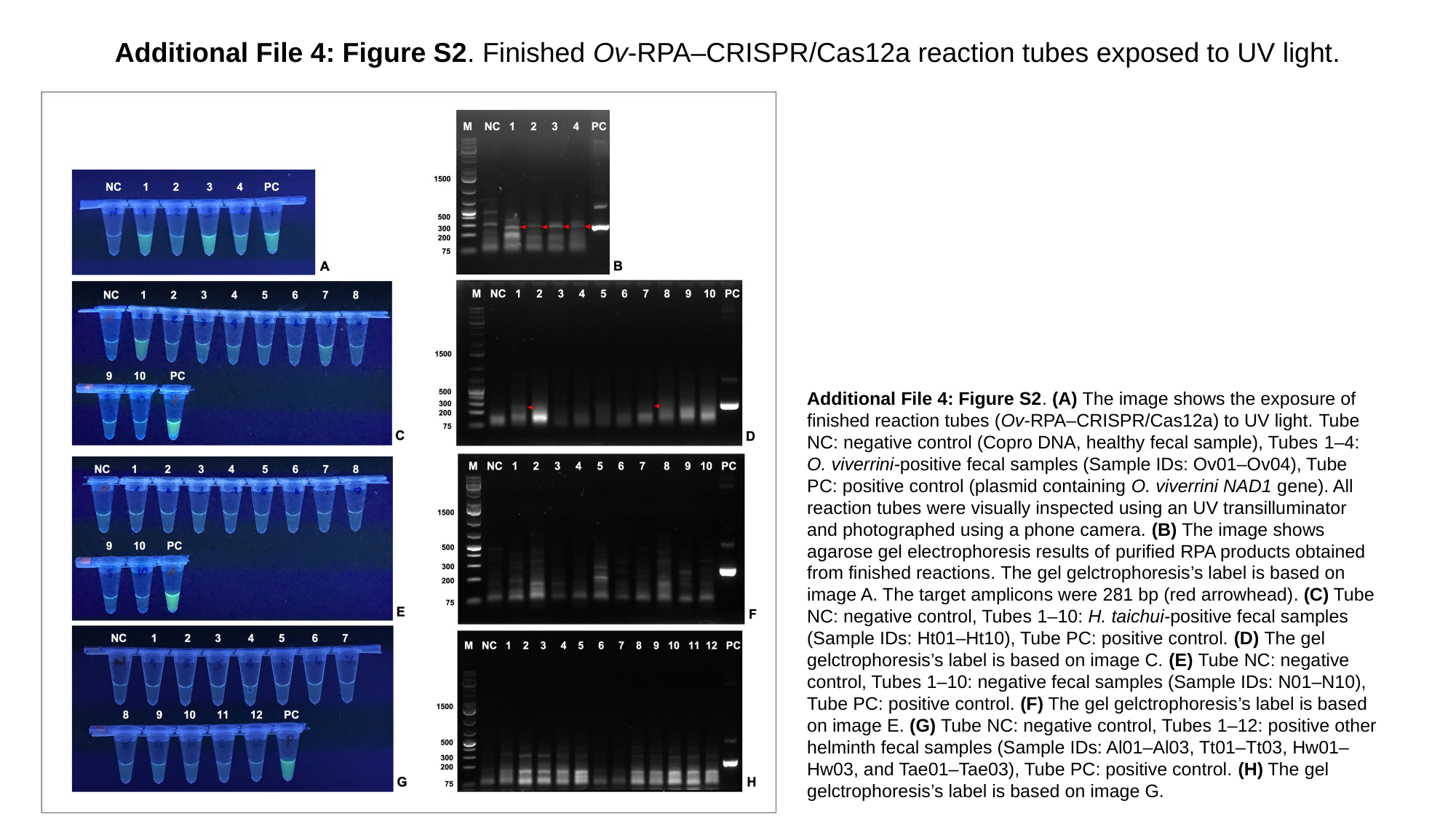

Additional File 4: Figure S2. Finished Ov-RPA–CRISPR/Cas12a reaction tubes exposed to UV light.
Additional File 4: Figure S2. (A) The image shows the exposure of finished reaction tubes (Ov-RPA–CRISPR/Cas12a) to UV light. Tube NC: negative control (Copro DNA, healthy fecal sample), Tubes 1–4: O. viverrini-positive fecal samples (Sample IDs: Ov01–Ov04), Tube PC: positive control (plasmid containing O. viverrini NAD1 gene). All reaction tubes were visually inspected using an UV transilluminator and photographed using a phone camera. (B) The image shows agarose gel electrophoresis results of purified RPA products obtained from finished reactions. The gel gelctrophoresis’s label is based on image A. The target amplicons were 281 bp (red arrowhead). (C) Tube NC: negative control, Tubes 1–10: H. taichui-positive fecal samples (Sample IDs: Ht01–Ht10), Tube PC: positive control. (D) The gel gelctrophoresis’s label is based on image C. (E) Tube NC: negative control, Tubes 1–10: negative fecal samples (Sample IDs: N01–N10), Tube PC: positive control. (F) The gel gelctrophoresis’s label is based on image E. (G) Tube NC: negative control, Tubes 1–12: positive other helminth fecal samples (Sample IDs: Al01–Al03, Tt01–Tt03, Hw01–Hw03, and Tae01–Tae03), Tube PC: positive control. (H) The gel gelctrophoresis’s label is based on image G.
