# Supplementary material for: Ov-RPA–CRISPR/Cas12a assay for the detection of Opisthorchis viverrini infection in field-collected human feces
Source: Parasit Vectors. 2024 Feb 21;17:80. doi: 10.1186/s13071-024-06134-7 (PMC10882828; doi:10.1186/s13071-024-06134-7)
Supplement: Supplementary file 5 — Additional file 5: Figure S3. Inverted gel electrophoresis image of Figure S2D. [file 13071_2024_6134_MOESM5_ESM.pptx]

## Slide 1
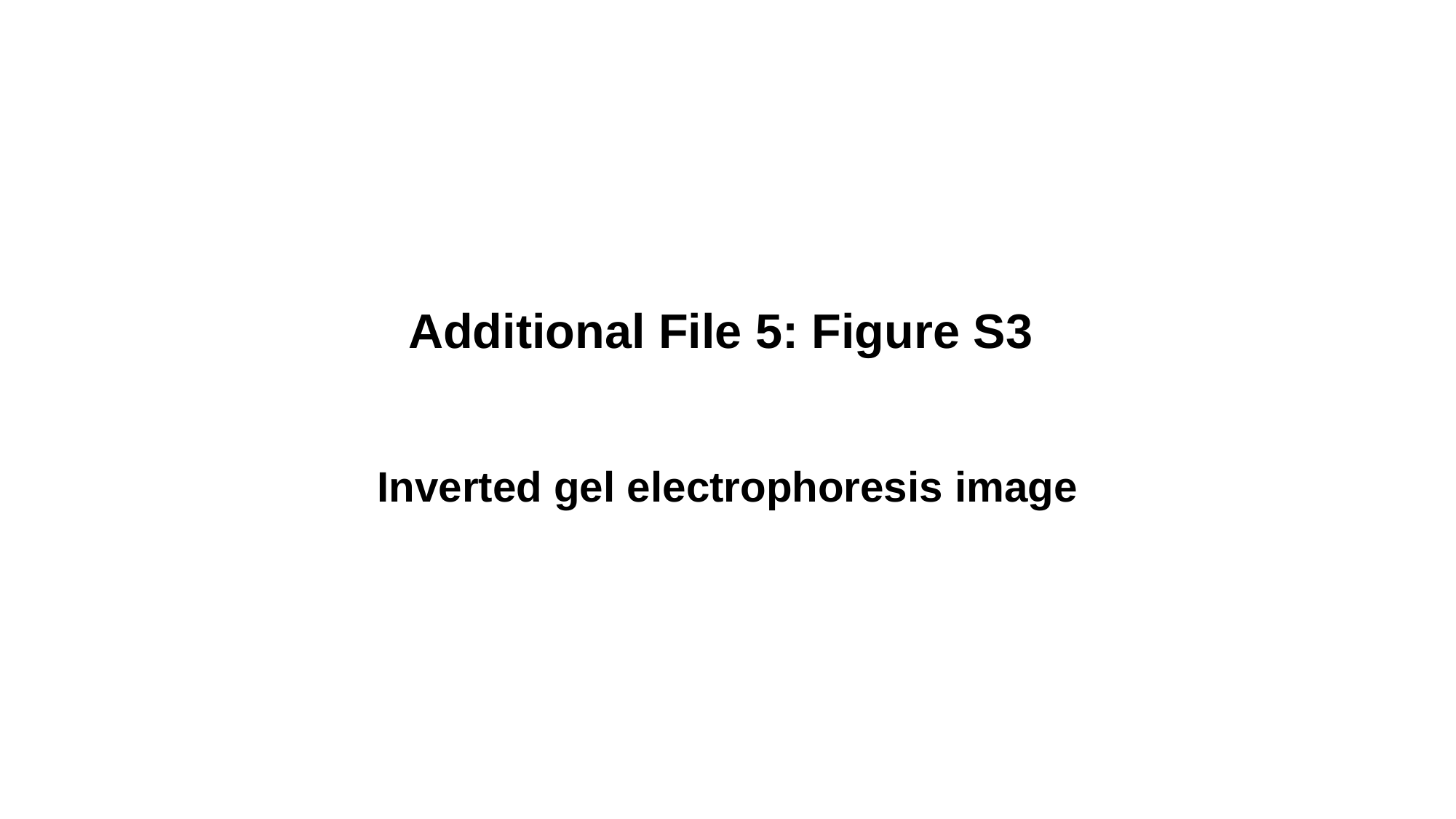

Additional File 5: Figure S3
Inverted gel electrophoresis image

## Slide 2
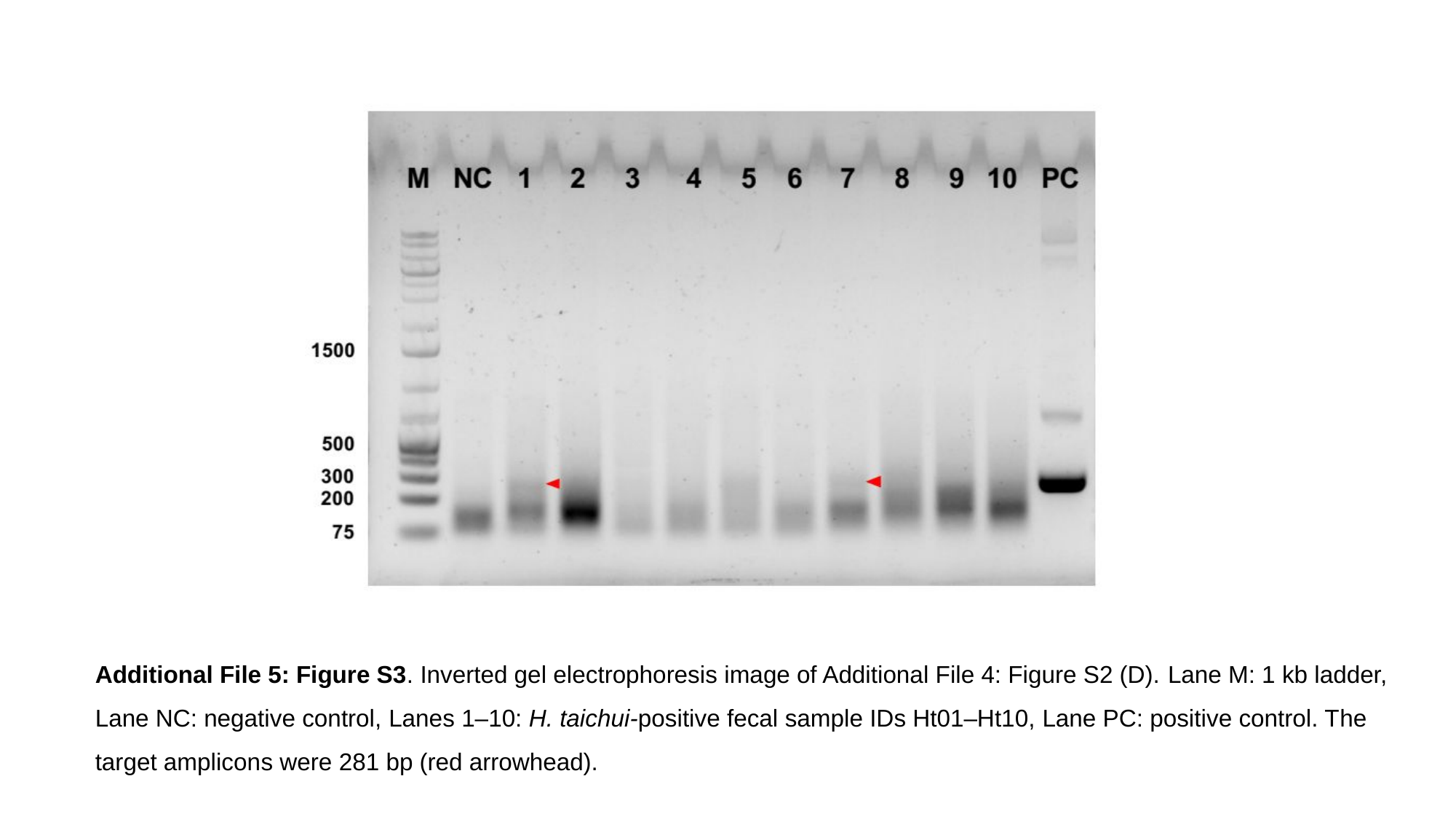

Additional File 5: Figure S3. Inverted gel electrophoresis image of Additional File 4: Figure S2 (D). Lane M: 1 kb ladder, Lane NC: negative control, Lanes 1–10: H. taichui-positive fecal sample IDs Ht01–Ht10, Lane PC: positive control. The target amplicons were 281 bp (red arrowhead).
