# Supplementary material for: Ov-RPA–CRISPR/Cas12a assay for the detection of Opisthorchis viverrini infection in field-collected human feces
Source: Parasit Vectors. 2024 Feb 21;17:80. doi: 10.1186/s13071-024-06134-7 (PMC10882828; doi:10.1186/s13071-024-06134-7)
Supplement: Supplementary file 7 — Additional file 7: Figure S4. Cutoff plots obtained from ROC curve analysis comparing the Ov-RPA–CRISPR/Cas12a assay and KK method for detecting O. viverrini infection in fecal samples. [file 13071_2024_6134_MOESM7_ESM.pptx]

## Slide 1
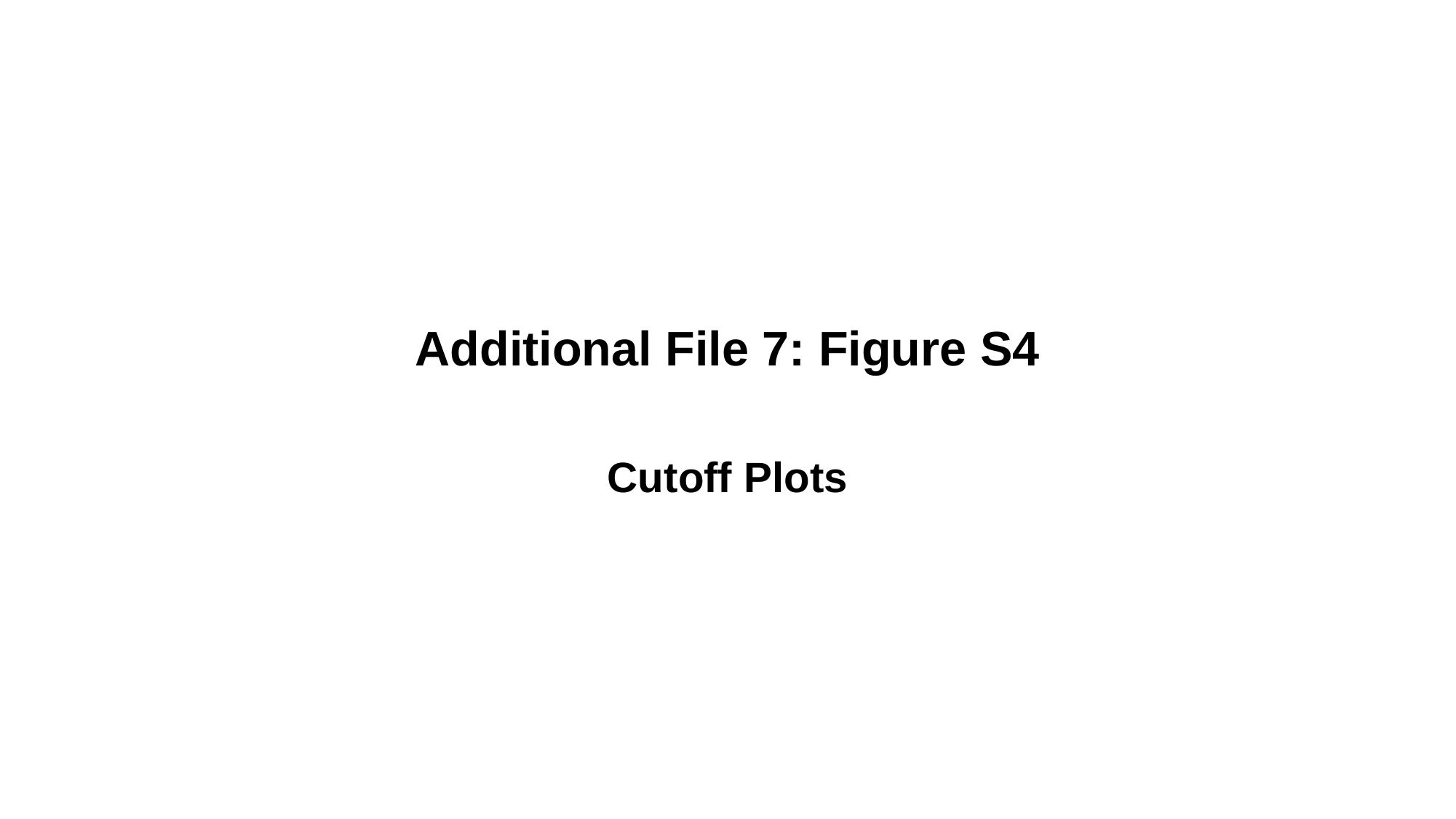

Additional File 7: Figure S4
Cutoff Plots

## Slide 2
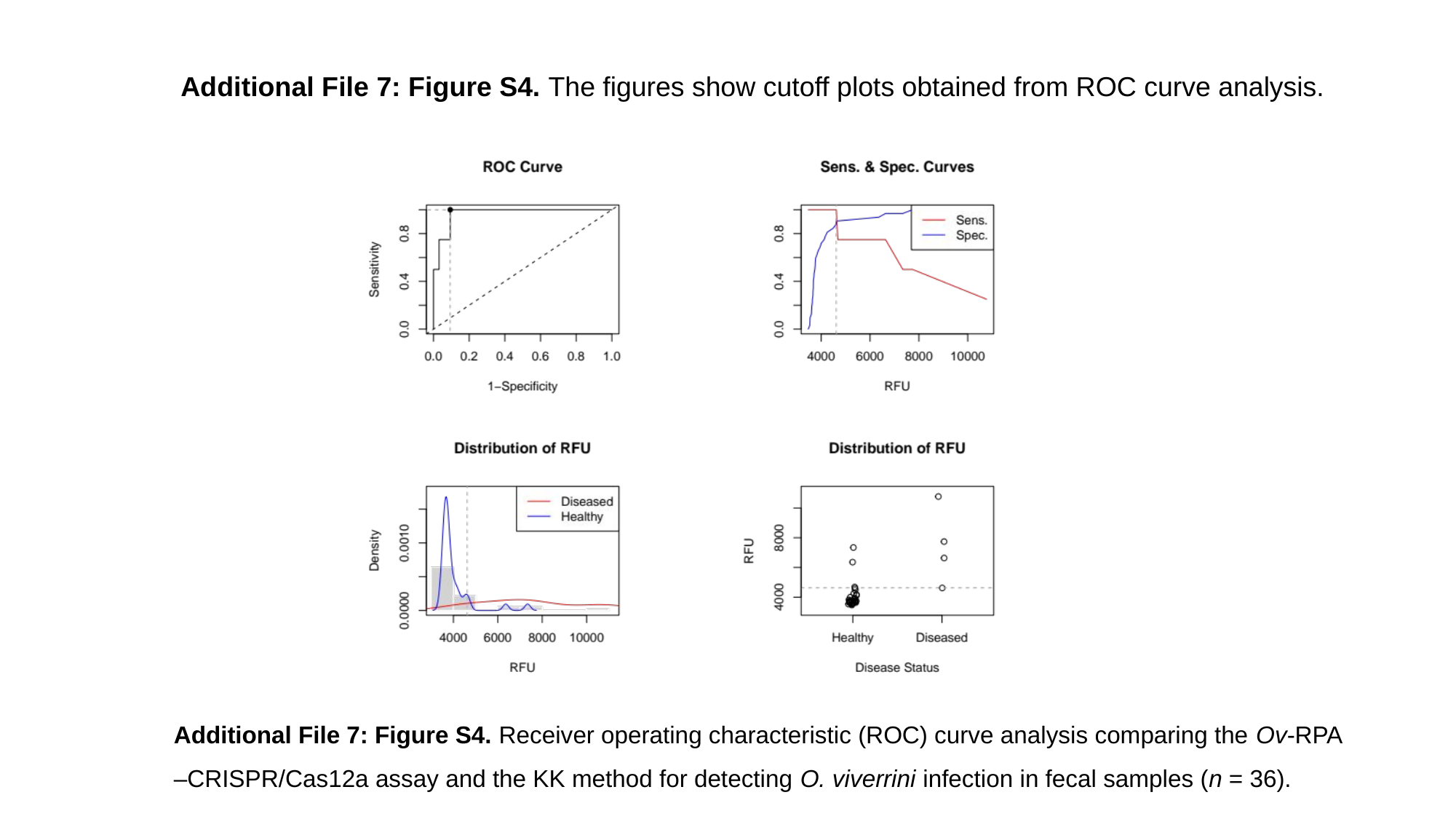

Additional File 7: Figure S4. The figures show cutoff plots obtained from ROC curve analysis.
Additional File 7: Figure S4. Receiver operating characteristic (ROC) curve analysis comparing the Ov-RPA –CRISPR/Cas12a assay and the KK method for detecting O. viverrini infection in fecal samples (n = 36).
