# Supplementary material for: Ov-RPA–CRISPR/Cas12a assay for the detection of Opisthorchis viverrini infection in field-collected human feces
Source: Parasit Vectors. 2024 Feb 21;17:80. doi: 10.1186/s13071-024-06134-7 (PMC10882828; doi:10.1186/s13071-024-06134-7)
Supplement: Supplementary file 9 — Additional file 9: Figure S5. Examples of RPA amplicons targeting human actin beta gene (hACTB) and pET20b+–GsCPC2 (GsCPC2). [file 13071_2024_6134_MOESM9_ESM.pptx]

## Slide 1
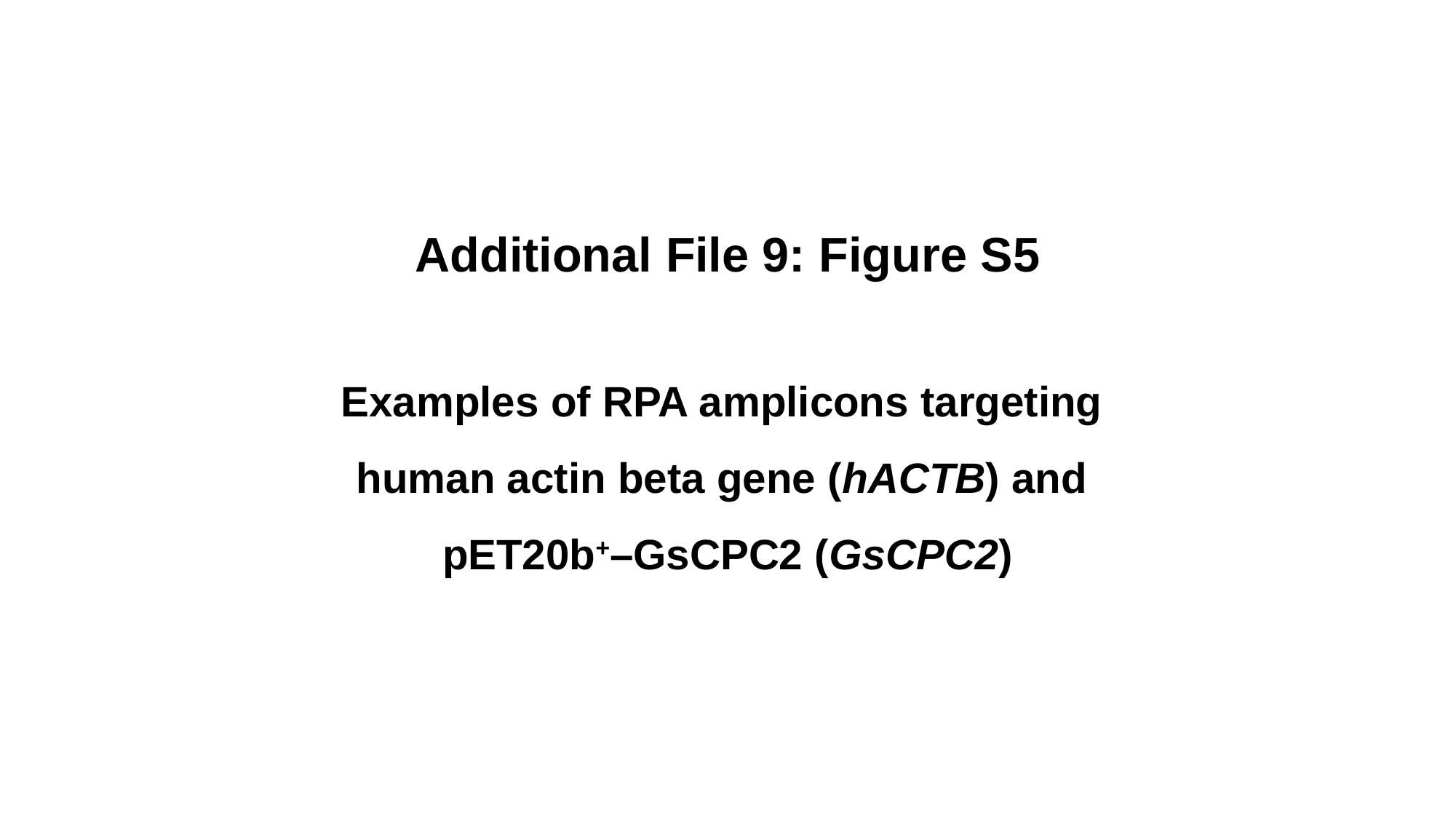

Additional File 9: Figure S5
Examples of RPA amplicons targeting
human actin beta gene (hACTB) and
pET20b+–GsCPC2 (GsCPC2)

## Slide 2
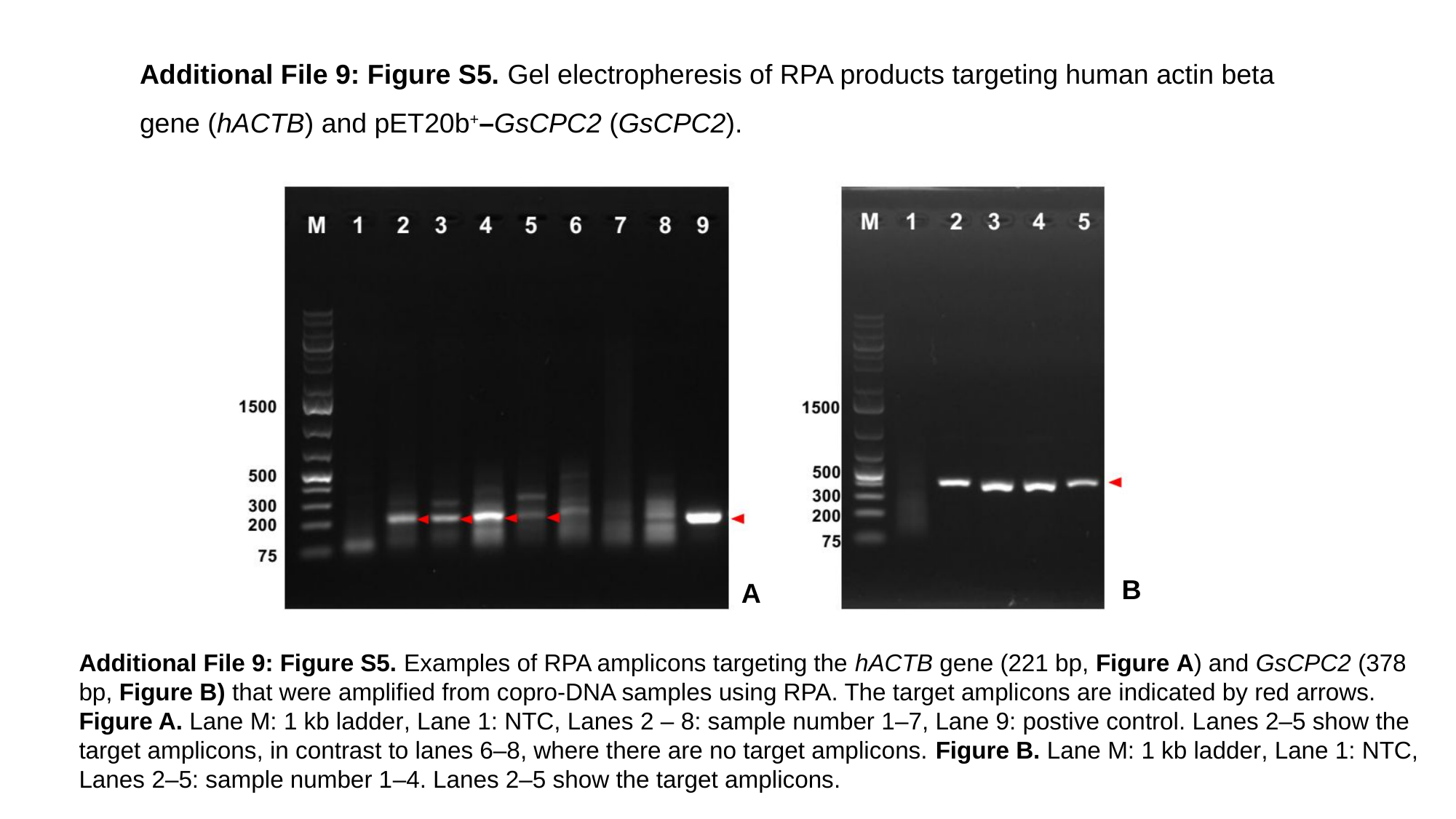

Additional File 9: Figure S5. Gel electropheresis of RPA products targeting human actin beta gene (hACTB) and pET20b+–GsCPC2 (GsCPC2).
B
A
Additional File 9: Figure S5. Examples of RPA amplicons targeting the hACTB gene (221 bp, Figure A) and GsCPC2 (378 bp, Figure B) that were amplified from copro-DNA samples using RPA. The target amplicons are indicated by red arrows.
Figure A. Lane M: 1 kb ladder, Lane 1: NTC, Lanes 2 – 8: sample number 1–7, Lane 9: postive control. Lanes 2–5 show the target amplicons, in contrast to lanes 6–8, where there are no target amplicons. Figure B. Lane M: 1 kb ladder, Lane 1: NTC, Lanes 2–5: sample number 1–4. Lanes 2–5 show the target amplicons.
