# Supplementary material for: Ov-RPA–CRISPR/Cas12a assay for the detection of Opisthorchis viverrini infection in field-collected human feces
Source: Parasit Vectors. 2024 Feb 21;17:80. doi: 10.1186/s13071-024-06134-7 (PMC10882828; doi:10.1186/s13071-024-06134-7)
Supplement: Supplementary file 11 — Additional file 11: Figure S6. Cutoff plots obtained from ROC curve analysis comparing Ov-RPA–CRISPR/Cas12a assay and standard methods (KK and/or FECT) for detecting O. viverrini infection in fecal samples. [file 13071_2024_6134_MOESM11_ESM.pptx]

## Slide 1
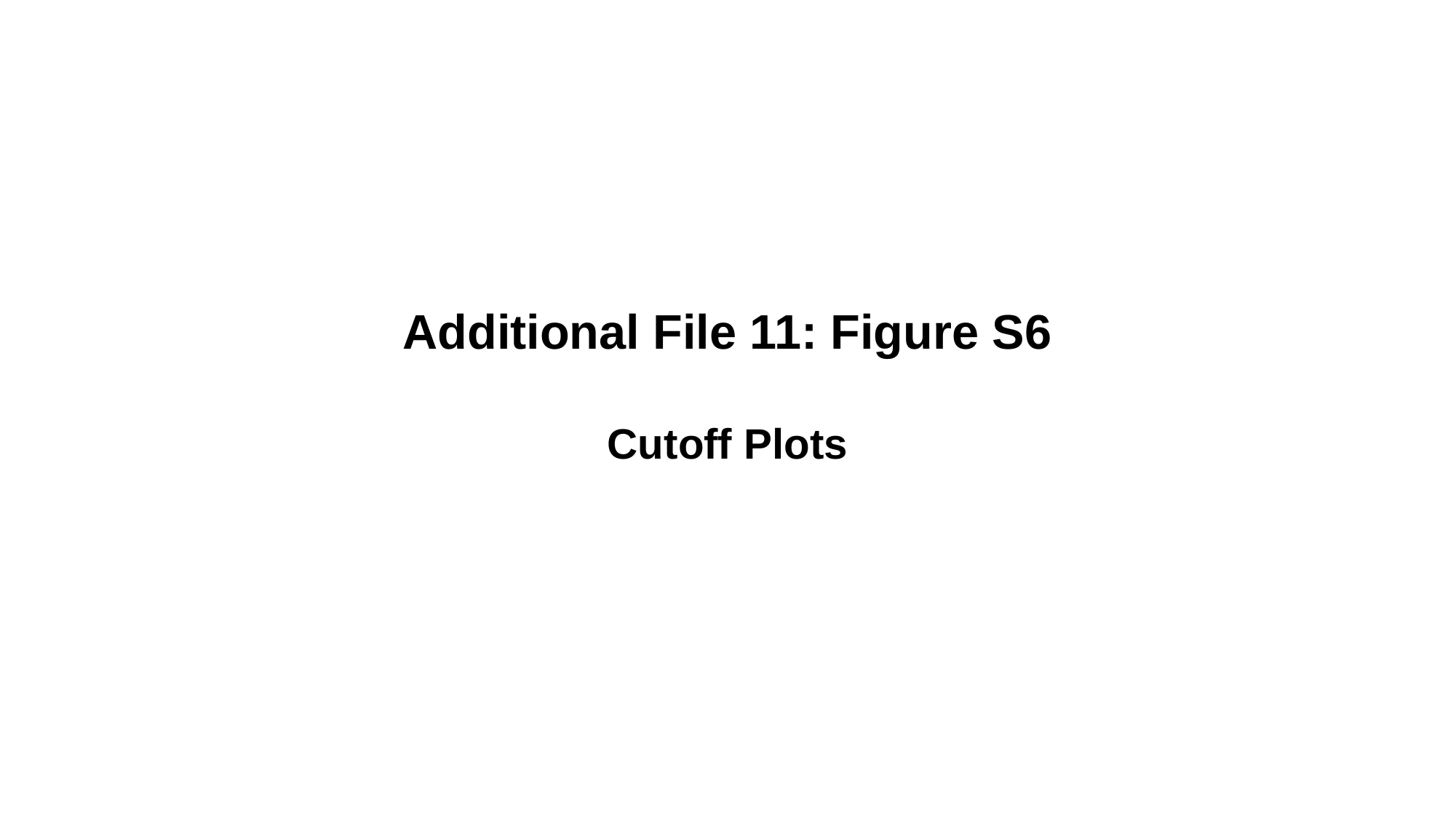

Additional File 11: Figure S6
Cutoff Plots

## Slide 2
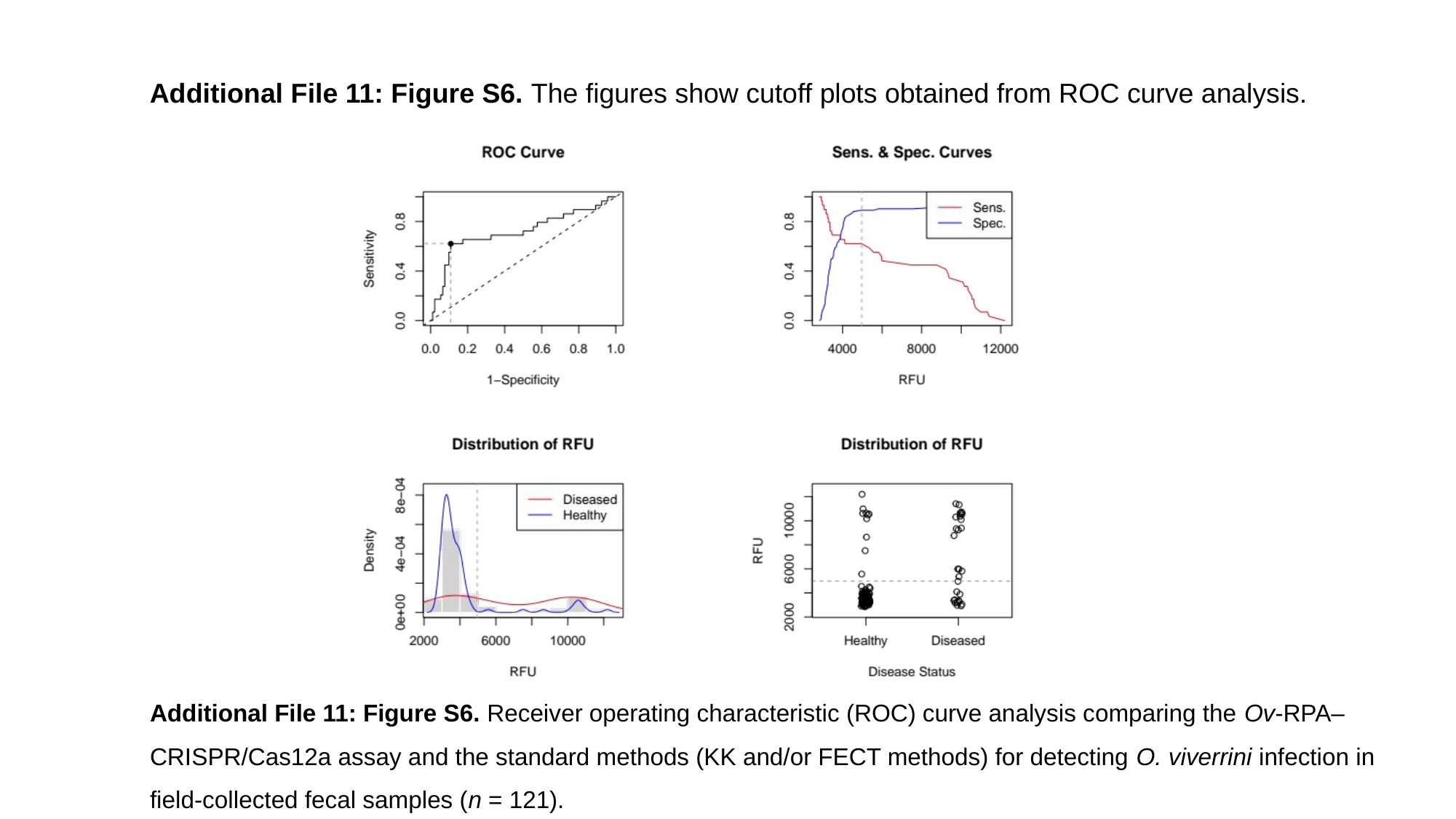

Additional File 11: Figure S6. The figures show cutoff plots obtained from ROC curve analysis.
Additional File 11: Figure S6. Receiver operating characteristic (ROC) curve analysis comparing the Ov-RPA–CRISPR/Cas12a assay and the standard methods (KK and/or FECT methods) for detecting O. viverrini infection in field-collected fecal samples (n = 121).
