# Supplementary material for: Ov-RPA–CRISPR/Cas12a assay for the detection of Opisthorchis viverrini infection in field-collected human feces
Source: Parasit Vectors. 2024 Feb 21;17:80. doi: 10.1186/s13071-024-06134-7 (PMC10882828; doi:10.1186/s13071-024-06134-7)
Supplement: Supplementary file 12 — Additional file 12: Figure S7. Examples of O. viverrini-like egg morphology. [file 13071_2024_6134_MOESM12_ESM.pptx]

## Slide 1
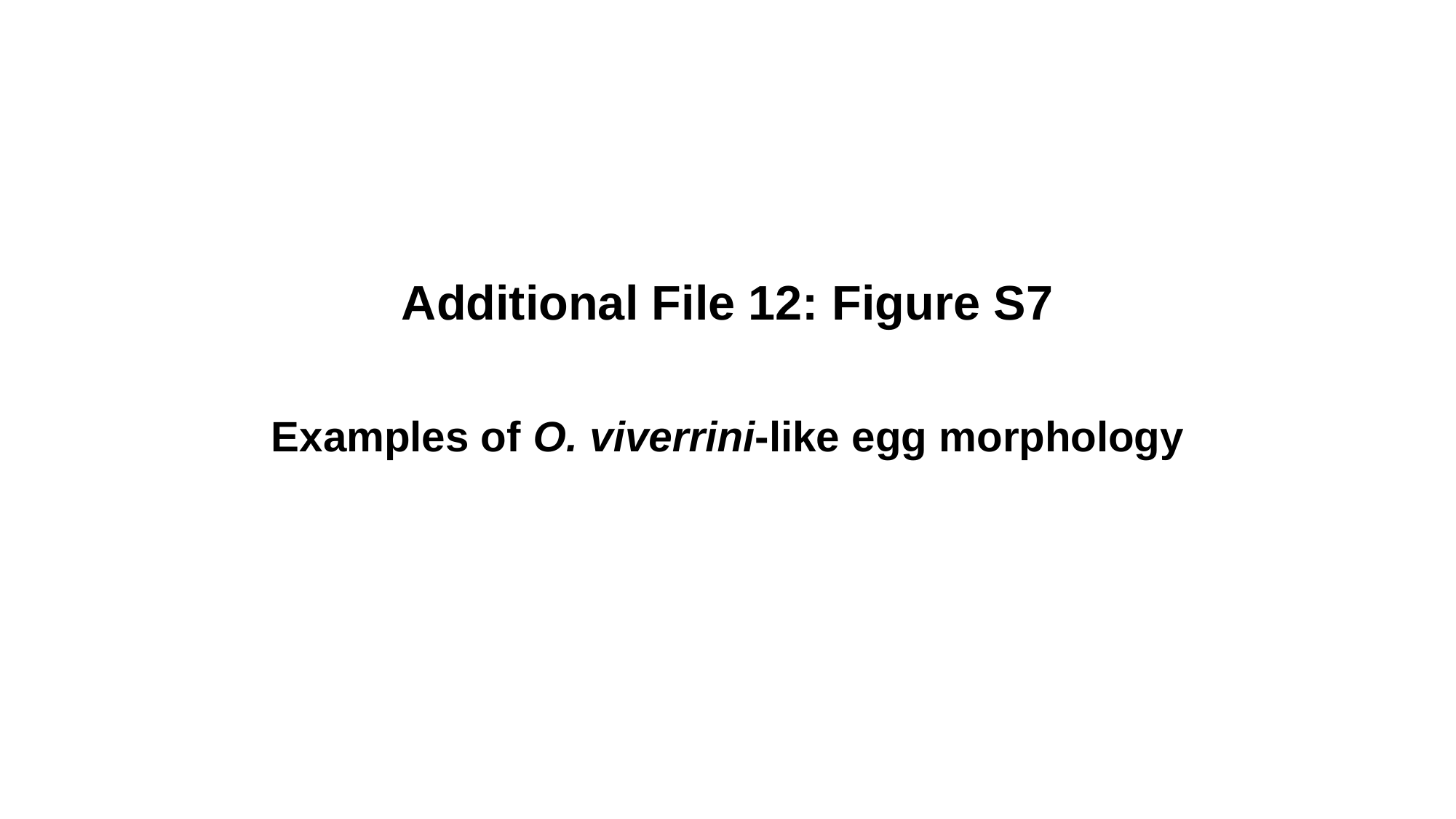

Additional File 12: Figure S7
Examples of O. viverrini-like egg morphology

## Slide 2
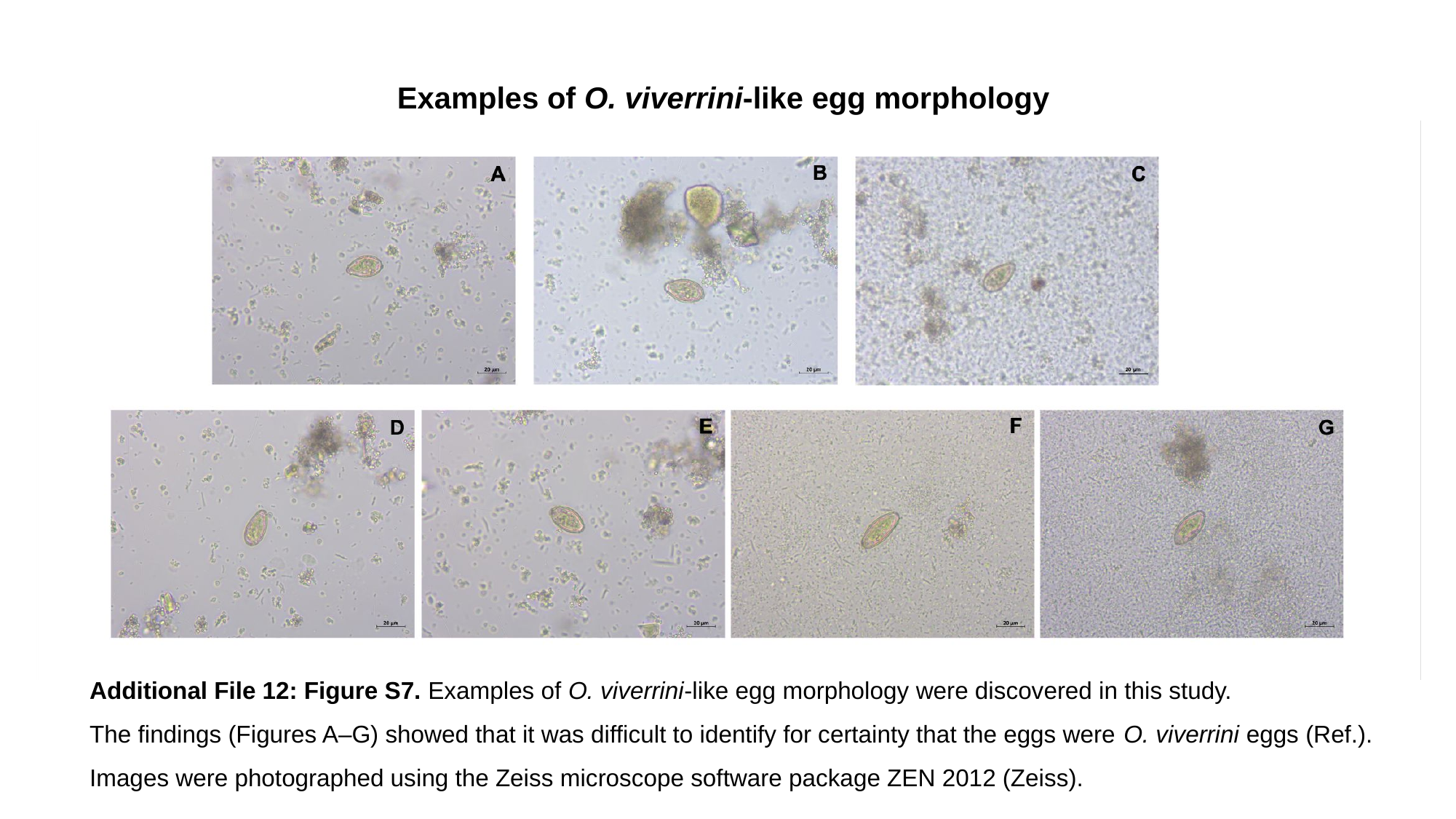

Examples of O. viverrini-like egg morphology
Additional File 12: Figure S7. Examples of O. viverrini-like egg morphology were discovered in this study.
The findings (Figures A–G) showed that it was difficult to identify for certainty that the eggs were O. viverrini eggs (Ref.). Images were photographed using the Zeiss microscope software package ZEN 2012 (Zeiss).

## Slide 3
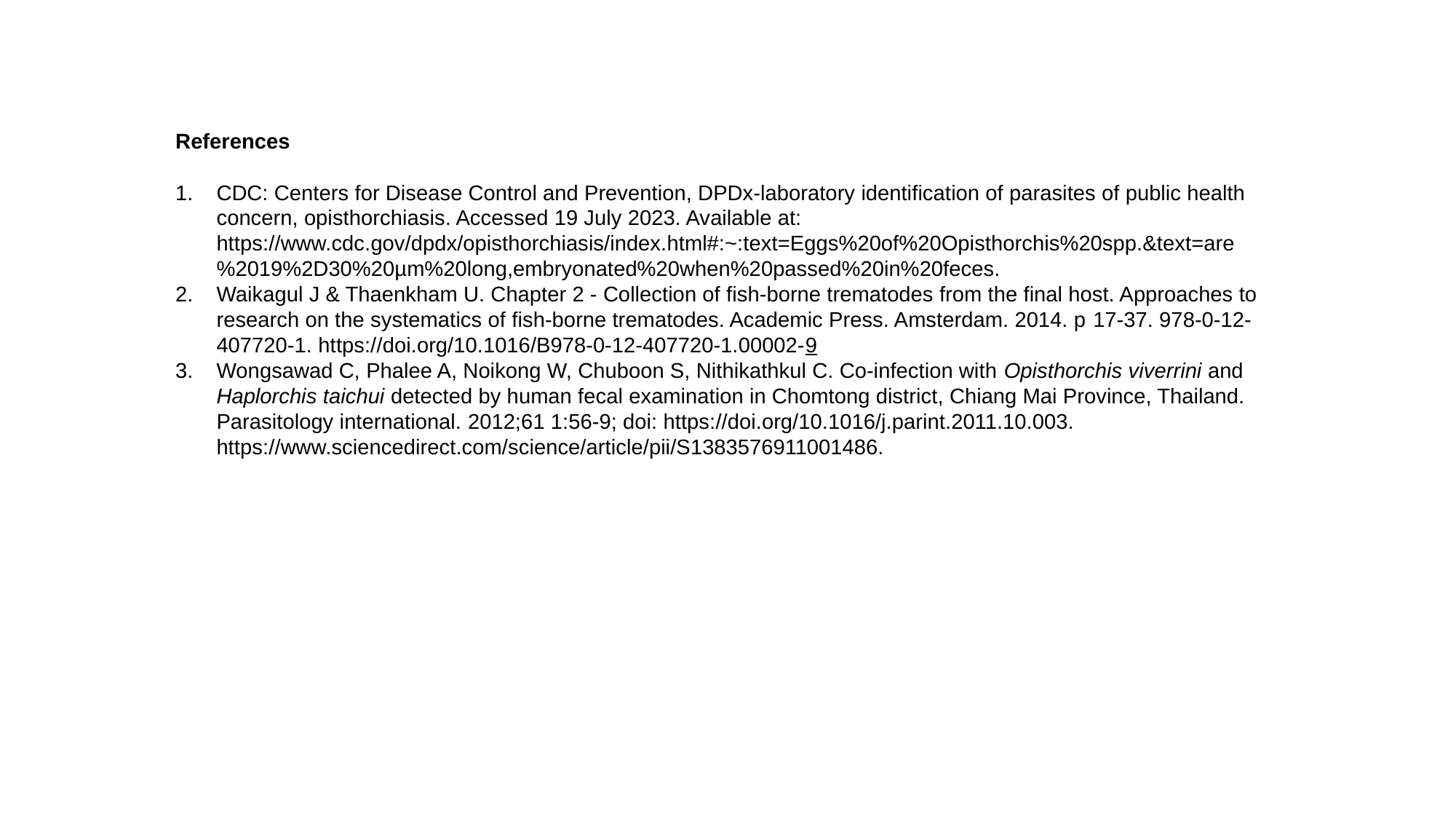

References
CDC: Centers for Disease Control and Prevention, DPDx-laboratory identification of parasites of public health concern, opisthorchiasis. Accessed 19 July 2023. Available at: https://www.cdc.gov/dpdx/opisthorchiasis/index.html#:~:text=Eggs%20of%20Opisthorchis%20spp.&text=are%2019%2D30%20µm%20long,embryonated%20when%20passed%20in%20feces.
Waikagul J & Thaenkham U. Chapter 2 - Collection of fish-borne trematodes from the final host. Approaches to research on the systematics of fish-borne trematodes. Academic Press. Amsterdam. 2014. p 17-37. 978-0-12-407720-1. https://doi.org/10.1016/B978-0-12-407720-1.00002-9
Wongsawad C, Phalee A, Noikong W, Chuboon S, Nithikathkul C. Co-infection with Opisthorchis viverrini and Haplorchis taichui detected by human fecal examination in Chomtong district, Chiang Mai Province, Thailand. Parasitology international. 2012;61 1:56-9; doi: https://doi.org/10.1016/j.parint.2011.10.003. https://www.sciencedirect.com/science/article/pii/S1383576911001486.
